# Multi-cohort validation of a lipid metabolism and ferroptosis-associated index for prognosis and immunotherapy response prediction in hormone receptor-positive breast cancer

**DOI:** 10.7150/ijbs.113213

**Published:** 2025-06-09

**Authors:** Cheng Zeng, Jiani Wang, Shen Zhao, Yuhan Wei, Yalong Qi, Shuning Liu, Yuanyi Wang, Hewei Ge, Xiaoqi Yang, Yujing Tan, Yizhou Jiang, Haili Qian, Fei Ma

**Affiliations:** 1 Department of Medical Oncology, National Cancer Center/National Clinical Research Center for Cancer/Cancer Hospital, Chinese Academy of Medical Sciences and Peking Union Medical College, Beijing, 100021, China.; 2 Key Laboratory of Breast Cancer in Shanghai, Department of Breast Surgery, Fudan University Shanghai Cancer Center, Shanghai Medical College, Fudan University, Shanghai 200032, China; Department of Oncology, Shanghai Medical College, Fudan University, Shanghai, 200032, China.; 3 State Key Laboratory of Molecular Oncology, National Cancer Center/National Clinical Research Center for Cancer/Cancer Hospital, Chinese Academy of Medical Sciences and Peking Union Medical College, Beijing, 100021, China.

**Keywords:** lipid metabolism, ferroptosis, immunotherapy, ACSL4, hormone receptor-positive breast cancer

## Abstract

**Background:** Hormone receptor-positive (HR+) breast cancer exhibits significant heterogeneity influenced by lipid metabolism and ferroptosis (LMF). While immune checkpoint inhibitors have shown promise in neoadjuvant therapy, as evidenced by the KEYNOTE-756 and CheckMate 7FL trials, identifying the optimal patient population remains challenging. This study aims to classify molecular clusters based on LMF-related genes and develop the LMF_index to predict prognosis and immunotherapy response in HR+ breast cancer.

**Methods:** Transcriptome and clinical data of HR+ breast cancer were obtained from the Cancer Genome Atlas and Gene Expression Omnibus databases. Unsupervised clustering based on prognostic LMF-related genes identified molecular clusters, followed by tumor mutational burden (TMB) and immune microenvironment (TME) analysis. The LMF_index was constructed using least absolute shrinkage and selection operator and multivariate Cox regression analyses and validated across multiple internal and external cohorts. Its predictive value for neoadjuvant immunotherapy efficacy was assessed using GSE173839. Validation at the transcriptomic level was conducted in the Shanghai cohort, while protein-level validation was performed using multiplex immunohistochemistry (mIHC) on a tissue microarray comprising 113 breast cancer samples. Spatial analyses further examined the distribution of key panel genes within the TME.

**Results:** Two molecular clusters were identified in this study. Cluster 1 exhibited higher TMB, tumor purity, and Ki-67, while Cluster 2 showed greater CD8+ T cells and elevated PD-1, PD-L1, and CTLA4 expression. The LMF_index, derived from a seven-gene panel (KRT5, CD209, KLRB1, MRC1, UGT2B4, FABP7, and BIRC3), effectively stratified patients into high and low LMF_index groups, with high LMF_index patients showing significantly shorter overall survival. Patients with a low LMF_index demonstrated elevated ACSL4 expression, enhanced immune activity, higher immunophenoscores, and increased pathological complete response rates following neoadjuvant immunotherapy, indicating a greater potential benefit from immunotherapy. The prognostic value of the LMF_index was validated at the transcriptomic level in the Shanghai cohort and at the protein level using mIHC on a tissue microarray. Spatial analysis further demonstrated KLRB1 enrichment in the tumor stroma, correlating with CD8+ T cell and M1 macrophage infiltration, and an enhanced response to immunotherapy.

**Conclusions:** This study identified distinct LMF-related molecular clusters in HR+ breast cancer with unique prognostic and immune characteristics. The LMF_index shows potential as a prognostic biomarker and a guide for immunotherapy strategies in HR+ breast cancer.

## 1. Introduction

Breast cancer is one of the most common malignancies worldwide. According to the global cancer statistics report, approximately 2.3 million new breast cancer cases were diagnosed in 2022, accounting for 11.6% of all cancers, making it the second most common cancer^1^. Breast cancer is primarily classified based on the expression of estrogen receptor (ER), progesterone receptor (PR), and human epidermal growth factor receptor 2 (HER2), into subtypes including hormone receptor-positive (HR+) breast cancer, HER2+ breast cancer, and triple-negative breast cancer (TNBC)^2^, ^3^. HR+ breast cancer is the most common subtype, accounting for 70% of all breast cancer cases^3^. Current standard treatment for HR+ breast cancer primarily relies on endocrine therapy, including selective estrogen receptor modulators (SERMs), aromatase inhibitors (AIs), and selective estrogen receptor degraders (SERDs)^4^, ^5^. In recent years, the addition of cyclin-dependent kinase 4/6 (CDK4/6) inhibitors to endocrine regimens has significantly improved progression-free survival (PFS) and overall survival (OS) in patients with advanced HR+ breast cancer^6^-^9^. Moreover, targeted therapies such as PI3K inhibitors and mTOR inhibitors are available for selected patients harboring PIK3CA mutations^10^, ^11^. Despite these advances, therapeutic resistance remains a major challenge, and most patients with advanced HR+ breast cancer eventually experience disease progression^12^, ^13^. Furthermore, not all patients are eligible for targeted therapies due to the absence of actionable genetic alterations, limiting the effectiveness of precision oncology in this population.

Given these therapeutic limitations, immunotherapy has emerged as a promising approach in HR+ breast cancer. Immune checkpoint inhibitors (ICIs), such as those targeting PD-1, PD-L1, or CTLA-4, have shown remarkable success in several cancer types by reactivating cytotoxic T cells to eliminate tumor cells^14^, ^15^. However, HR+ breast cancer is generally characterized by a "cold" immune phenotype with low tumor-infiltrating lymphocytes and limited PD-L1 expression, contributing to the relatively poor responsiveness to ICIs observed in this subtype^16^, ^17^. Nevertheless, recent clinical trials have provided new insights into the potential of ICIs in HR+ breast cancer. The phase III KEYNOTE-756 and CheckMate 7FL studies demonstrated that combining ICIs with neoadjuvant chemotherapy significantly improved the pathological complete response (pCR) rate in ER+/HER2- breast cancer patients^18^, ^19^. However, the observed increase in pCR was only approximately 10%, highlighting the limited overall benefit of ICIs in this subtype. The lack of reliable predictive biomarkers poses a significant challenge in optimizing patient selection and maximizing therapeutic outcomes. Since the efficacy of ICIs is closely linked to the tumor microenvironment (TME), a comprehensive characterization of the immune landscape and the development of robust biomarkers are essential for stratifying HR+ breast cancer patients. Identifying the subset most likely to benefit from ICIs will enhance the precision and efficacy of immunotherapy in this population.

Lipid metabolism plays a crucial role in the initiation and progression of tumors^20^, ^21^. Lipid metabolic processes such as fatty acid synthesis, oxidation, and cholesterol metabolism are closely linked to the development of breast cancer, and disturbances in lipid metabolism are associated with immune escape and drug resistance^22^-^24^. Ferroptosis, a distinct form of programmed cell death, is tightly linked to lipid metabolism, with fatty acid accumulation inducing oxidative stress and triggering cell death^25^. Conversely, the occurrence of ferroptosis may alter the metabolic state of tumor cells, influencing lipid metabolism processes^26^. Moreover, recent studies suggest that ferroptosis can influence immune cell recruitment and function within the TME, potentially impacting the effectiveness of immunotherapies^27^. Given the close interplay among lipid metabolism, ferroptosis, and immune regulation, we hypothesized that an integrative analysis of genes associated with lipid metabolism and ferroptosis (LMF) may yield a more robust framework for characterizing the immune landscape and identifying molecular clusters in HR+ breast cancer. In recent years, some researchers have utilized either lipid metabolism or ferroptosis-related genes to evaluate tumor prognosis and predict immunotherapy response^28^-^31^. However, their combined impact in HR+ breast cancer has not been systematically elucidated. Therefore, integrating LMF-related genes into a unified analytical model may deepen our understanding of the TME, and enhance the development of personalized immunotherapeutic strategies.

**Figure 1:**
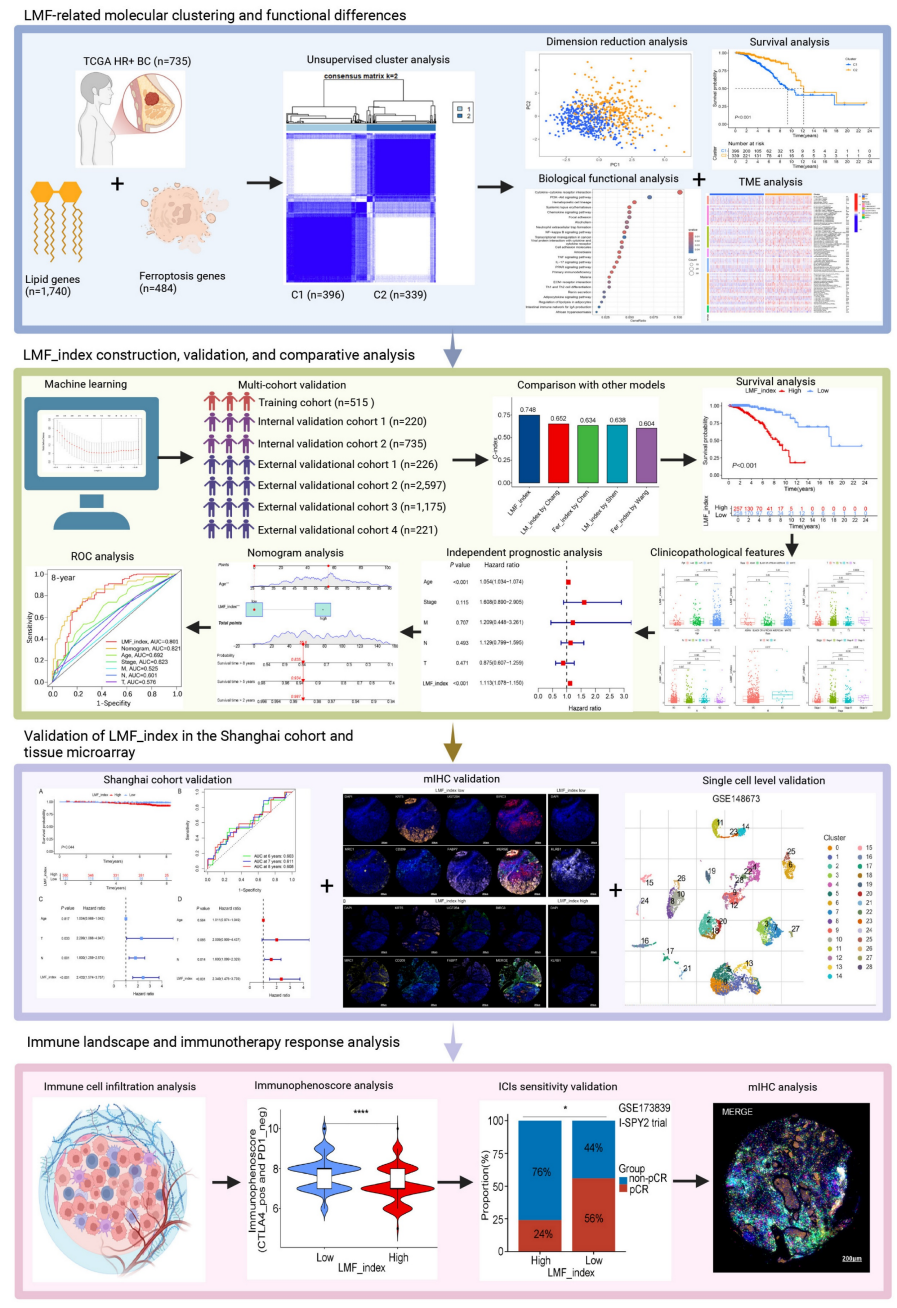
Flowchart of the study design.

In this study, we combined LMF-related genes for unsupervised clustering analysis, classifying HR+ breast cancer patients into two molecular clusters. We then constructed and validated the LMF_index across multiple cohorts to evaluate the prognosis of HR+ breast cancer patients. Furthermore, we explored the potential value of the LMF_index in reflecting TME features and predicting the efficacy of immunotherapy. Lastly, we validated the prognostic ability of the LMF_index using the Shanghai cohort and multiplex immunohistochemistry (mIHC) on breast cancer tissue microarrays. Further analysis revealed that high expression of KLRB1 is associated with increased infiltration of CD8+ T cells and M1 macrophages, as well as an improved response to immunotherapy. These findings offer new insights into prognosis prediction and immunotherapy for HR+ breast cancer. The study design is depicted in **Figure 1**.

## 2. Materials and Methods

### 2.1. Data collection and preprocessing

Transcriptomic data and corresponding clinical information for the Cancer Genome Atlas-breast invasive carcinoma (TCGA-BRCA) cohort were downloaded from the TCGA GDC database (https://portal.gdc.cancer.gov/) in January 2024. The dataset initially contained 1,097 breast cancer tissue samples. After excluding 105 samples with OS of less than 30 days or missing survival status, 241 TNBC and HER2+ samples, and 16 samples with incomplete clinical staging, 735 HR+ breast cancer samples were included in this study. Among them, 485 patients had documented records of receiving endocrine therapy. HR+ breast cancer was defined as ER and/or PR expression greater than 1% by immunohistochemistry. The 735 HR+ samples were randomly divided into a training cohort (n=515) and internal validation cohort 1 (n=220) using a 7:3 split with seed-based random assignment^32^. The GSE159956 dataset includes 295 breast cancer samples, of which 226 were HR+ samples. The GSE7390 dataset includes 198 breast cancer samples, with 134 HR+ samples. To increase the sample size for external validation, the gene expression matrices of 226 HR+ samples from GSE159956 and 134 HR+ samples from GSE7390 were batch-corrected and merged using the combat algorithm to form external validation cohort 1 using the sva package (version 3.54.0)^33^. The GSE96058 dataset contains 3,069 breast cancer samples. After removing 100 duplicate samples and 372 TNBC and HER2+ samples, 2,597 HR+ samples were included as external validation cohort 2. Among these, 2,141 patients had documented records of receiving endocrine therapy. Transcriptomic data and clinical information for the METABRIC dataset were downloaded from the cBioPortal database (https://www.cbioportal.org/) in January 2024. The METABRIC dataset includes 2,509 breast cancer samples, of which 528 were excluded due to missing survival time or status, and 806 TNBC or HER2+ samples were also removed, leaving 1,175 HR+ samples for external validation cohort 3. Among these, 856 patients had documented records of receiving endocrine therapy. The GSE173839 dataset contains 50 HR+ breast cancer patients who received neoadjuvant immunotherapy. A total of 484 ferroptosis-related genes were obtained from the FerrDb database (http://www.zhounan.org/ferrdb/current/) in December 2023 (**Table S1**). A total of 1,740 lipid metabolism-related genes used in this study were obtained from our previously published work (**Table S2**)^34^.

### 2.2. Unsupervised clustering analysis of LMF-related genes

In this study, differential expression analysis of LMF-related genes between HR+ breast cancer and normal tissues was performed using the limma package (version 3.62.1)^35^. The selection criteria for differential expression genes (DEGs) were set at |log2FC| > 1 with an adjusted *P*-value < 0.05^36^. Next, a univariate Cox regression analysis was conducted on these DEGs to assess their prognostic value. Genes with a *P*-value < 0.01 were selected for unsupervised clustering analysis using the ConsensusClusterPlus package (version 1.70.0)^37^. The consensus cumulative distribution function (CDF) under various cluster numbers was used to evaluate cluster stability and determine the optimal number of clusters. Principal component analysis (PCA) and t-distributed stochastic neighbor embedding (t-SNE) were employed to visualize the distribution characteristics of the different clusters^38^. OS and disease-free survival (DFS) of patients between two clusters were analyzed based on survival time and status.

### 2.3. Biological functional analysis between LMF-related molecular clusters

To investigate the biological functional differences between molecular clusters classified based on LMF-related genes, we first assessed the tumor mutational burden (TMB) and the overall mutational landscape between the two clusters. DEGs between the molecular clusters were identified using the limma package (version 3.62.1), with the threshold set at |log2FC| > 1 and an adjusted *P*-value < 0.05^35^, ^36^. Functional enrichment analyses, including Gene Ontology (GO) enrichment analysis and Kyoto Encyclopedia of Genes and Genomes (KEGG) pathway analysis, were performed to elucidate the biological processes and pathways associated with these DEGs. To evaluate differences in the TME, the ESTIMATE algorithm was applied to compare stromal scores, immune scores, and overall ESTIMATE scores between the molecular clusters^39^. Furthermore, key immune checkpoint molecules, including PD-1, PD-L1, and CTLA4, as well as estrogen receptor alpha (ERα), Ki67, and components of the PI3K/AKT/mTOR signaling pathway, were analyzed to assess their expression differences across the clusters. A comprehensive assessment of immune cell infiltration was conducted using multiple computational algorithms, including TIMER, CIBERSORT, CIBERSORT-ABS, QUANTISEQ, MCPcounter, xCell, and EPIC, ensuring a robust characterization of immune landscape variations between the clusters^40^, ^41^. In addition, hematoxylin and eosin (H&E) staining was performed on HR+ breast cancer tissue samples to analyze lymphocyte infiltration levels^42^, further validating the computational immune profiling results.

### 2.4. Construction of a prognostic LMF_index and comparison with existing indices

To construct a prognostic LMF_index and evaluate its predictive performance, we first conducted univariate Cox regression analysis on the DEGs between the two molecular clusters. To minimize overfitting, prognosis-related genes identified from the univariate analysis were further subjected to least absolute shrinkage and selection operator (LASSO) regression analysis using the glmnet package (version 4.1.8)^43^. The optimal *λ* value was determined via 10-fold cross-validation to achieve the best predictive performance for LMF-related genes^32^, ^36^. Subsequently, multivariate Cox regression analysis was performed to refine the LMF-related gene panel and determine their corresponding regression coefficients. An LMF_index was then calculated based on the selected genes using the following formula^36^, ^38^, ^44^:



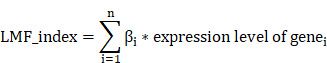



The relationships among LMF_index groups, molecular clusters, and survival outcomes were illustrated using Sankey diagrams. To assess the prognostic value of the LMF_index, we compared its predictive performance with existing indices, including the ferroptosis-related index by Wang *et al.*^28^, the ferroptosis-related index by Chen *et al.*^29^, the lipid metabolism-related index by Chang *et al.*^30^, and the lipid metabolism-related index by Shen *et al.*^31^. To ensure consistency with the literature, gene expression levels corresponding to each index were extracted, and multivariate Cox regression analysis was applied to obtain the respective regression coefficients for each gene^45^. The indices for each patient were then calculated. The predictive performance and clinical utility of the LMF_index were evaluated using concordance index (C-index), receiver operating characteristic (ROC) curve analysis, and survival analyses, providing a comprehensive comparison with previously established prognostic indices^45^.

### 2.5. Internal and external validation of the LMF_index

The performance of the LMF_index was first assessed in the training cohort, while two internal validation cohorts (internal validation 1 and internal validation 2) were employed for internal validation of the LMF_index. To increase the sample size for external validation, we merged the GSE159956 and GSE7390 datasets after batch effect correction to form external validation 1. In addition, external validation 2 (GSE96058), external validation 3 (METABRIC dataset), and external validation 4 (Kaplan-Meier Plotter) were used for independent external validation of the LMF_index. First, a scatter plot was generated in the training cohort to visualize the relationship between the LMF_index and survival status of HR+ breast cancer patients. Kaplan-Meier survival analysis was then conducted to compare OS between LMF_index low and LMF_index high groups. Furthermore, ROC curves were plotted to evaluate the sensitivity and specificity of the LMF_index for predicting 2-year, 5-year, and 8-year survival rates in patients with HR+ breast cancer. The same analyses were performed in the two internal validation cohorts and the three external validation cohorts. In external validation 4 (Kaplan-Meier Plotter), OS and recurrence-free survival (RFS) were analyzed in ER-positive breast cancer patients.

### 2.6. Clinical subgroup survival analysis based on the LMF_index

To further evaluate the performance of the LMF_index across different clinical subgroups, we conducted Kaplan-Meier survival analysis stratified by clinicopathological characteristics of HR+ breast cancer patients. The subgroups analyzed included age (≤40 years, 40-70 years, ≥70 years), race (White, Asian, Black or African American), tumor stage (T1, T2, T3, T4), nodal status (N0, N1, N2, N3), metastatic status (M0, M1), and overall disease stage (Stage I, Stage II, Stage III, Stage IV).

### 2.7. Independent prognostic analysis and nomogram construction for the LMF_index

To investigate prognostic factors in HR+ breast cancer patients, both univariate and multivariate Cox regression analyses were performed, incorporating clinicopathological characteristics and the LMF_index. Additionally, a nomogram combining clinicopathological characteristics and the LMF_index was constructed to predict patient prognosis^38^. The predictive accuracy of the nomogram was evaluated using the C-index. Furthermore, ROC curve analysis was performed to assess the predictive performance of the nomogram score, LMF_index, and clinical factors (age, overall disease stage, T stage, N stage, M stage) for 2-year, 5-year, and 8-year survival outcomes in HR+ breast cancer patients^32^.

### 2.8. Association between the LMF_index and the TME landscape

To explore the TME landscape in HR+ breast cancer, multiple algorithms, including XCELL, TIMER, QUANTISEQ, MCPCOUNTER, CIBERSORT-ABS, and CIBERSORT, were utilized to assess the spearman correlation between the LMF_index and the levels of immune cell infiltration, and bubble plots were generated to visualize these relationships^40^, ^41^. Subsequently, the ESTIMATE algorithm was applied to analyze the stromal score, immune score, and total ESTIMATE score for LMF_index high and LMF_index low groups^39^. Additionally, the CIBERSORT method and single-sample gene set enrichment analysis (ssGSEA) were conducted to evaluate differences in immune cell infiltration levels and the activity of immune-related pathways between different LMF_index populations^46^, ^47^.

### 2.9. Evaluation of immunotherapy sensitivity based on the LMF_index

To evaluate the clinical relevance of the LMF_index in predicting ICIs response, spearman correlation analysis was first conducted to assess its association with immune checkpoint-related gene expression. Then, immunophenoscores (IPS) for TCGA-HR_BRCA patients were obtained from the Cancer Immunome Atlas (TCIA) (http://tcia.at/). IPS was compared between the LMF_index high and LMF_index low groups, with higher IPS indicating greater sensitivity to ICIs^32^, ^36^. To validate the predictive value of the LMF_index in immunotherapy response, data from the GSE173839 dataset (I-SPY2 clinical trial) were analyzed. Pathological complete response (pCR) rates were compared between LMF_index high and LMF_index low groups following neoadjuvant immunotherapy.

### 2.10. Analysis of expression levels of LMF_index panel genes

To assess the expression patterns of LMF_index panel genes in normal breast tissue and breast cancer tissue, we utilized data from the University of Alabama at Birmingham Cancer data analysis portal (UALCAN) (https://ualcan.path.uab.edu/analysis.html). Additionally, to explore the expression profiles of LMF_index panel genes at the single-cell level, we employed the Tumor Immune Single-cell Hub 2 (TISCH2) (http://tisch.comp-genomics.org/home/), a comprehensive single-cell RNA sequencing (scRNA-seq) database focusing on the TME. Specifically, the GSE148673 dataset was used to analyze the LMF_index panel genes from a single-cell perspective, providing insights into its expression levels in tumor cells and immune cells.

### 2.11. Patients and tissue samples

The tissue microarrays used in this study, which consisted of tumor samples from 136 breast cancer patients, were procured from Shanghai Outdo Biotech Company in full compliance with the relevant regulations. The study was conducted in accordance with the ethical principles outlined in the Declaration of Helsinki. Written informed consent was obtained from all participants, ensuring voluntary participation. The use of human tumor tissue samples was approved by the Ethics Committee of Shanghai Outdo Biotech Company (approval number: YBM-05-01, YBM-05-02).

### 2.12. Multiplex immunohistochemistry staining and image acquisition

The mIHC staining protocol was validated by the EACRI IHC Core at the Providence Cancer Institute (Portland, OR), and the methods have been previously reported^48^. This study utilized consecutive sections from a tissue microarray. One section was stained for KRT5, UGT2B4, BIRC3, MRC1, CD209, and FABP7, while the other section was stained for PanCK, CD8, KLRB1, PD-L1, CD68, and CD163. An additional section was stained for PanCK, CD8, ACSL4, and GPX4 to evaluate the spatial distribution of ferroptosis-related proteins and immune cell infiltration within the epithelial and stromal compartments. Briefly, the tissue microarray slides were baked at 63°C for 1 hour, followed by deparaffinization using a fully automatic staining machine (LEICAST5020, LEICA). Antigen retrieval was performed after deparaffinization. Endogenous peroxidase activity was quenched using commercial hydrogen peroxide for 10 minutes. The microarrays were blocked for 10 minutes before being incubated for 1 hour with one of the following primary antibodies to perform mIHC staining: anti-CD68 (dilution: 1:500, Abcam), anti-CD8 (dilution: 1:500, Abcam), anti-CD163 (dilution: 1:300, Abcam), anti-PD-L1 (dilution: 1:200, Abcam), anti-PanCK (dilution: 1:200, Abcam), anti-KLRB1 (dilution: 1:50, Abcam), anti-KRT5 (dilution: 1:1000, Proteintech), anti-MRC1 (dilution: 1:200, Abcam), anti-FABP7 (dilution: 1:50, Abcam), anti-BIRC3 (dilution: 1:400, Abcam), anti-UGT2B4 (dilution: 1:50, Abcam), anti-CD209 (dilution: 1:50, Abcam), anti-ACSL4 (dilution: 1:100, Abcam), and anti-GPX4 (dilution: 1:100, Abcam). After washing the slides with TBST buffer, secondary antibody incubation was carried out using ready-to-use secondary antibodies (SM802, DAKO) for 10 minutes. Subsequently, Opal dye (Opal 7-color Manual IHC Kit, NEL801001KT, PerkinElmer) was applied and incubated at room temperature for 10 minutes. Microwave treatment was employed to remove the antibody complex between each cycle of staining. These steps were repeated until all markers in the panel were stained. Slides were counterstained with DAPI for 5 minutes and mounted using antifade mounting medium.

Multispectral imaging of the slides was conducted using the Tissue-FAXS system (TissueFAXS Spectra, TissueGnostics GmbH, Vienna, Austria). The acquired image data were imported into Strata-Quest analysis software (version 7.1.129, TissueGnostics GmbH, Vienna, Austria) for further processing. Spectral unmixing was performed to separate single-channel fluorescence signals, with the DAPI channel utilized for nuclear identification. A distance radius was defined based on the staining patterns of each protein channel, with the nucleus serving as a reference, enabling the detection of specific protein fluorescence signals. Thresholds were set according to the staining patterns of each channel, allowing for the identification and quantification of positively stained cell populations. The number and intensity of cells exhibiting single, double, or multiple positive staining were recorded for subsequent analysis.

### 2.13. Statistical analysis

Statistical analyses were performed using R software (version 4.3.0) and GraphPad Prism (version 8.2.1). Differences between categories were assessed using the Wilcoxon test for continuous variables and the Chi-square test for categorical variables. OS was compared across various categories using Kaplan-Meier curves, with statistical significance evaluated via the log-rank test. To identify independent prognostic factors, univariate and multivariate Cox regression analyses were performed. The predictive ability of the LMF_index was assessed using ROC curves and nomograms. For comparisons between two groups, continuous variables were analyzed using the t-test (for normally distributed data) or the Mann-Whitney U test (for non-normally distributed data). A *P*-value of <0.05 was considered statistically significant. The following significance thresholds were used: *P < 0.05; **P < 0.01; ***P < 0.001; ****P < 0.0001.

## 3. Results

### 3.1. Molecular clustering based on LMF-related genes in HR+ breast cancer

**Figure 2:**
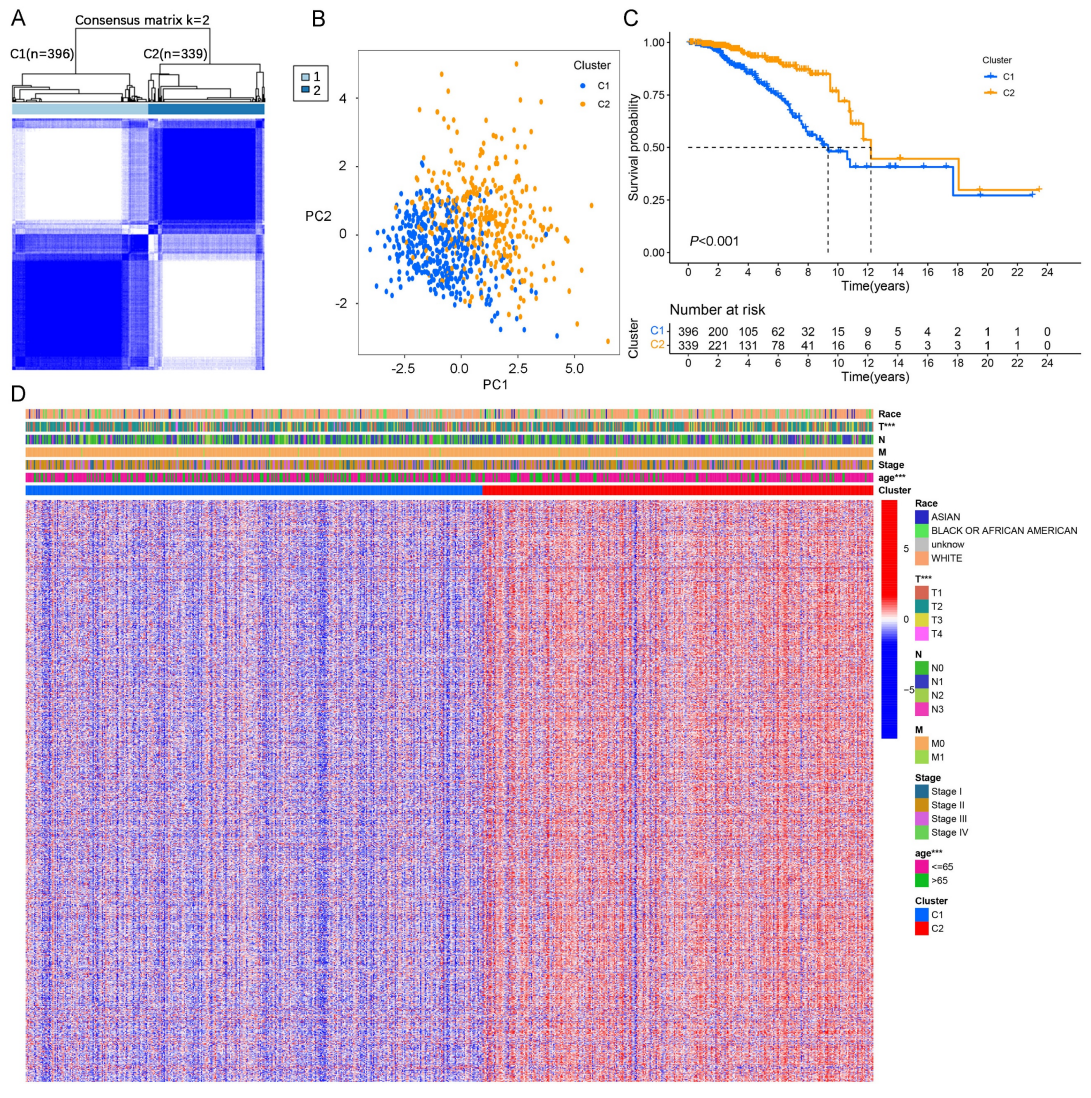
** Molecular clusters of LMF-related genes in HR+ breast cancer. (A)** Consensus matrix of unsupervised clustering analysis. **(B)** Principal Component Analysis (PCA) plots showing distinct separation between the two identified molecular clusters. **(C)** Kaplan-Meier survival curves comparing overall survival (OS) between the two molecular clusters. (**D**) Heatmap illustrating gene expression profiles and clinicopathological characteristics across the two molecular clusters. ***P < 0.001.

In this study, we identified 422 DEGs associated with LMF (**Figure S1A and Table S3**). Univariate Cox regression analysis further revealed 13 DEGs significantly associated with prognosis in HR+ breast cancer (*P* < 0.01, **Figure S1B**). Correlation heatmap analysis and protein-protein interaction (PPI) network demonstrated strong associations among TNFSF4, TP63, CD24, ESR1, and PRLR, suggesting their potential roles in HR+ breast cancer progression (**Figure S1C-D**). To classify molecular clusters, we performed unsupervised clustering analysis, stratifying patients into two distinct molecular clusters: cluster 1 (n=396) and cluster 2 (n=339) (**Figure 2A and Figure S2A**). PCA and tSNE demonstrated a clear separation between cluster 1 and cluster 2, confirming the robustness and stability of the molecular classification derived from ConsensusClusterPlus analysis (**Figure 2B and Figure S2B**). Kaplan-Meier survival analysis revealed that patients in cluster 2 exhibited significantly longer OS and DFS compared to those in cluster 1 (both *P* < 0.001, **Figure 2C and Figure S2C**). Furthermore, a heatmap of gene expression profiles highlighted significant differences in expression patterns between the two molecular clusters (**Figure 2D**). In summary, these findings demonstrate that HR+ breast cancer can be classified into two distinct molecular clusters based on LMF-related gene expression, with cluster 2 exhibiting a more favourable prognosis compared to cluster 1.

### 3.2. Biological functional differences between the two LMF-related molecular clusters

To elucidate the biological mechanisms underlying the prognostic differences between the two molecular clusters, we first assessed TMB levels in cluster 1 and cluster 2. The results demonstrated that patients in cluster 1 exhibited significantly higher TMB compared to those in cluster 2 (**Figure S3A**). Mutation analysis revealed that genes such as CDH1, MYH9, and UBR5 displayed higher mutation frequencies in cluster 1, whereas GATA3, CSMD2, and TG exhibited higher mutation rates in cluster 2 (**Figure S3B**). To further explore the functional differences between the two molecular clusters, differential gene expression analysis identified 1,102 DEGs (adjusted *P* < 0.05, **Table S4**). GO enrichment analysis revealed that these DEGs were significantly involved in production of molecular mediators of immune response, T cell receptor complex, receptor-ligand activity, and cytokine activity (**Figure S3C**). KEGG pathway analysis showed enrichment in cytokine-cytokine receptor interaction, PI3K-AKT signaling pathway, and NF-kappa B signaling pathway (**Figure S3D**), suggesting potential differences in the immune microenvironment between the two molecular clusters.

**Figure 3:**
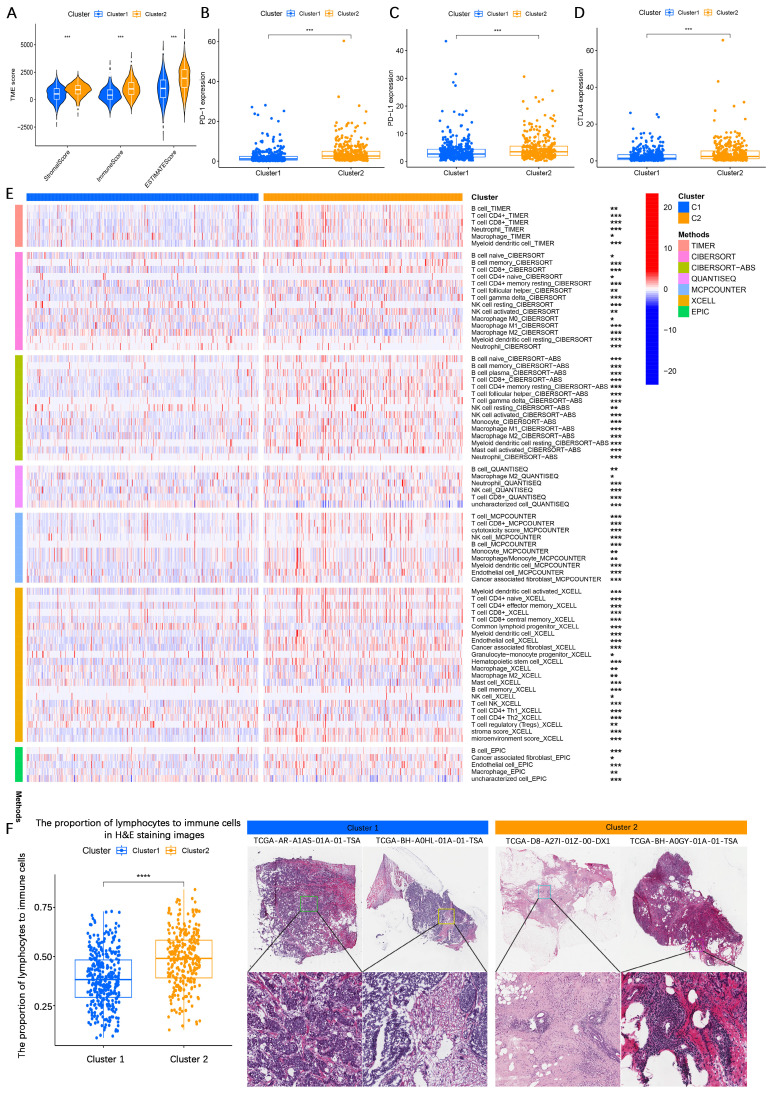
** Biological functional analysis of LMF-related molecular clusters in HR+ breast cancer. (A)** Analysis of stromal score, immune score, and ESTIMATE score between the two clusters using the ESTIMATE algorithm. The comparison indicates differences in the tumor microenvironment (TME) composition. **(B-D)** Differential expression analysis of immune checkpoint molecules PD-1, PD-L1, and CTLA4 between the two molecular clusters. The variation in expression suggests potential differences in immune evasion mechanisms and responses to immunotherapy. **(E)** Immune cell infiltration analysis comparing the two molecular clusters based on multiple algorithms, providing a comprehensive overview of immune cell infiltration patterns. **(F)** Hematoxylin and eosin (H&E) staining comparison across different molecular clusters, indicating more lymphocytic infiltration in cluster 2. *P < 0.05; **P < 0.01; ***P < 0.001; ****P < 0.0001.

To further evaluate differences in TME composition, the ESTIMATE algorithm was applied to assess immune and stromal components based on gene expression data. The analysis demonstrated that patients in cluster 1 had significantly lower stromal score, immune score, and ESTIMATE score compared to those in cluster 2 (**Figure 3A**), indicating higher tumor purity in cluster 1 and greater immune cell infiltration in cluster 2. Additionally, immune checkpoint expression analysis revealed significantly higher expression levels of PD-1, PD-L1, and CTLA-4 in cluster 2 compared to cluster 1 (**Figure 3B-D**). Moreover, patients in cluster 1 exhibited significantly higher expression of PI3K, AKT, mTOR, ER, and Ki-67 compared to those in cluster 2, suggesting more active proliferative signaling in cluster 1 (**Figures S3E-I**). To further characterize immune infiltration patterns, multiple methods were employed. A heatmap analysis demonstrated that cluster 2 tumors exhibited significantly higher immune cell infiltration than cluster 1 tumors (**Figure 3E**). This finding was corroborated by H&E staining, which showed increased lymphocyte infiltration in cluster 2 tumor tissues compared to cluster 1 (**Figure 3F**).

Collectively, these findings suggest that HR+ breast cancer patients in cluster 1 exhibit higher tumor purity, more aggressive malignant characteristics, and lower immune infiltration, whereas patients in cluster 2 display greater immune cell infiltration and upregulated immune checkpoint expression, which may contribute to a relatively better prognosis.

### 3.3. Construction and validation of the LMF_index and comparison with existing indices

**Figure 4:**
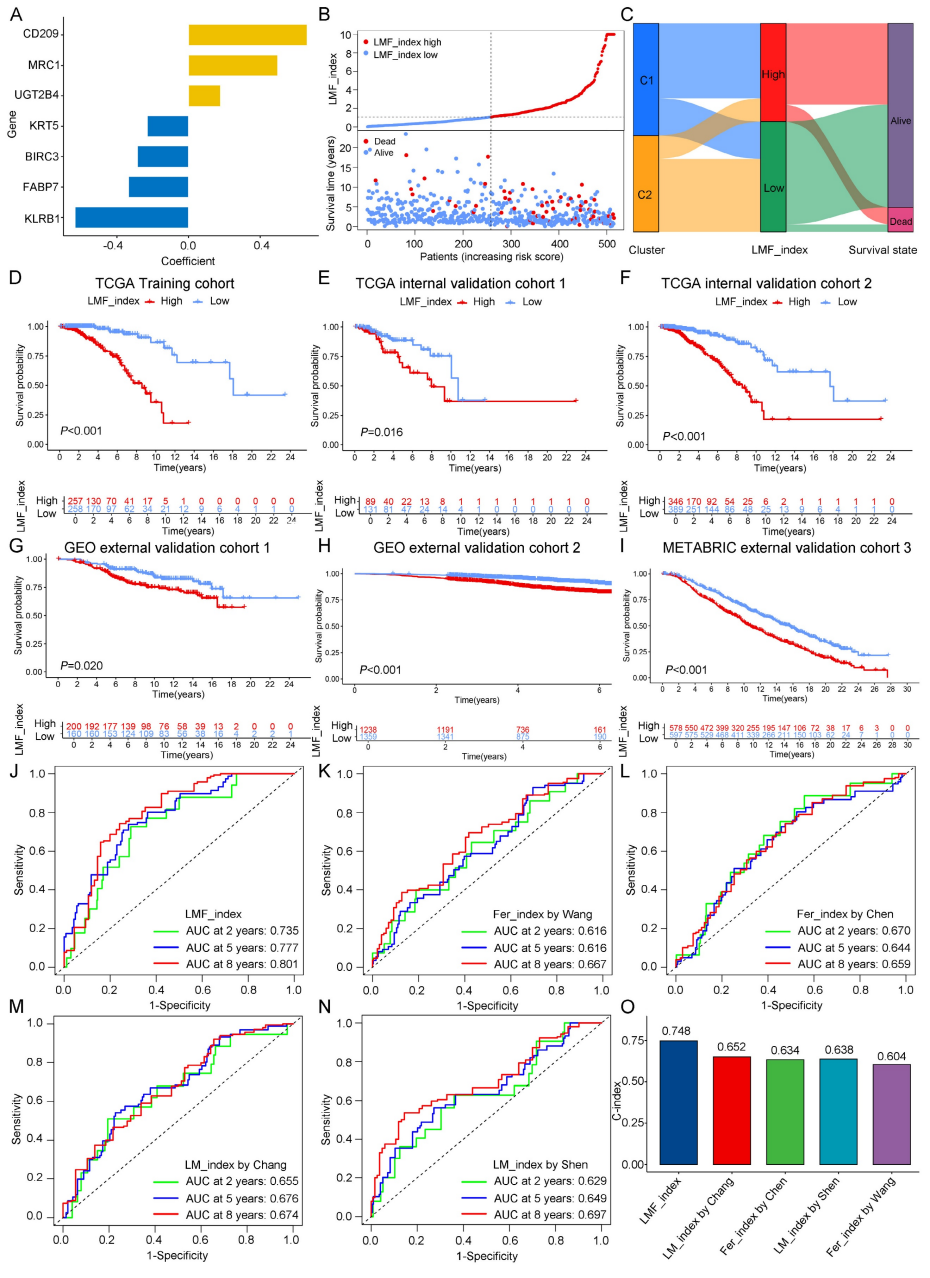
** Construction and validation of the LMF_index and comparison with existing indices. (A)** LMF_index panel genes and their corresponding regression coefficients identified by multivariate Cox regression analysis. **(B)** Distribution of LMF_index and scatter plot of survival status.** (C)** Sankey diagram depicting the relationships among molecular clusters, LMF_index, and survival status, providing a visual overview of patient classification and outcomes. **(D-I)** Kaplan-Meier survival curves for the TCGA training cohort, internal validation cohorts and external validation cohorts. **(J-N)** Time-dependent receiver operating characteristic (ROC) curve analysis comparing the area under the curve (AUC) values at 2, 5, and 8 years for the LMF_index and existing indices. **(O)** Comparison of the concordance index (C-index) across different indices, demonstrating the overall predictive performance of each index in terms of concordance with actual outcomes.

Given the significant prognostic differences observed between the two molecular clusters based on LMF-related genes, we sought to develop a robust prognostic index (LMF_index) for HR+ breast cancer. To achieve this, we conducted univariate Cox regression analysis on the 1,102 DEGs identified between the two clusters, leading to the selection of 55 genes significantly associated with prognosis (**Table S5**). To assess the robustness of the LMF_index, the HR+ breast cancer cohort (n = 735) was randomly divided into a training cohort (n = 515, **Table S7**) and a testing cohort (n = 220, **Table S8**) at a 7:3 ratio. The training cohort was used to construct the LMF_index. Subsequently, LASSO regression analysis was performed to prevent overfitting, using the optimal *λ* value, which refined the gene panel to 14 genes (**Figure S4A-B and Table S6**). Further refinement through multivariate Cox regression analysis identified seven key genes: KRT5, CD209, KLRB1, MRC1, UGT2B4, FABP7, and BIRC3 (**Figure 4A**). The LMF_index was then constructed based on the expression levels of these genes and their corresponding regression coefficients, formulated as follows: LMF_index = (-0.23034 × KRT5) + (0.66262 × CD209) + (-0.63397 × KLRB1) + (0.49821 × MRC1) + (0.17847 × UGT2B4) + (-0.33535 × FABP7) + (-0.28564 × BIRC3). Patients were subsequently stratified into LMF_index high and LMF_index low groups based on the median LMF_index. In the training cohort, a scatter plot of the risk index and survival status revealed that patients with a high LMF_index had a higher death rate (**Figure 4B**). Further analysis revealed that patients with a high LMF_index were predominantly from cluster 1, with significantly higher LMF_index compared to those in cluster 2 (**Figure 4C and Figure S4C**). Kaplan-Meier survival analysis demonstrated significantly shorter OS in LMF_index high patients compared to LMF_index low patients (*P* < 0.001, **Figure 4D**). These findings were consistently replicated in both internal validation cohorts (internal validation cohorts 1 and 2) and external validation cohorts (external validation cohorts 1, 2, and 3), where LMF_index high patients exhibited significantly worse OS outcomes (**Figure 4E-I**). In external validation cohort 4, LMF_index high patients also demonstrated poorer recurrence-free survival (RFS) in addition to lower OS (**Figure S4D**).

To assess the predictive performance of the LMF_index, we compared it with previously established indices, including ferroptosis_index (Wang *et al.*, Chen *et al.*) and lipid metabolism_index (Chang *et al.*, Shen *et al.*). To ensure comparability, all analyses were performed across the HR+ breast cancer cohort. Kaplan-Meier survival analysis revealed that patients classified as low index group by all five indices exhibited significantly better OS (**Figure S4E**). The ROC curve analysis showed that the LMF_index had superior predictive accuracy, with area under the curve (AUC) values of 0.735, 0.777, and 0.801 for 2-, 5-, and 8-year survival, respectively (**Figure 4J**). While the ferroptosis_index and lipid metabolism_index also exhibited AUC values above 0.6 (**Figure 4K-N**), they were consistently lower than those of the LMF_index. Moreover, the C-index of the LMF_index was 0.748, outperforming all other ferroptosis_indices and lipid metabolism_indices (**Figure 4O**). Additionally, all genes in the LMF_index panel—except UGT2B4—showed a positive correlation with ferroptosis scores, as well as with the activity of fatty acid oxidation and fatty acid synthesis pathways (**Figure S5**).

To further validate the prognostic utility of the LMF_index across different clinical pathological subgroups, Kaplan-Meier survival analyses were performed. The results indicated that, except for N3, M1, and Stage IV subgroups, LMF_index high patients had significantly shorter OS compared to LMF_index low patients (**Figure S6**). Additionally, the distribution of LMF_index across various clinical characteristics was examined. The findings demonstrated that patients aged over 70 had a significantly higher LMF_index than those under 70. Similarly, patients with T4-stage tumors exhibited a higher LMF_index than those with T1, T2, or T3-stage disease. Patients with distant metastasis (M1) and those with Stage IV disease had significantly higher LMF_index values than their M0 and earlier-stage counterparts, respectively (**Figure S7**). In summary, the LMF_index effectively stratifies HR+ breast cancer patients into prognostic subgroups, demonstrating superior predictive accuracy for OS compared to existing indices, with validation across multiple cohorts and clinical subgroups.

### 3.4. Independent prognostic analysis of the LMF_index and nomogram construction

**Figure 5:**
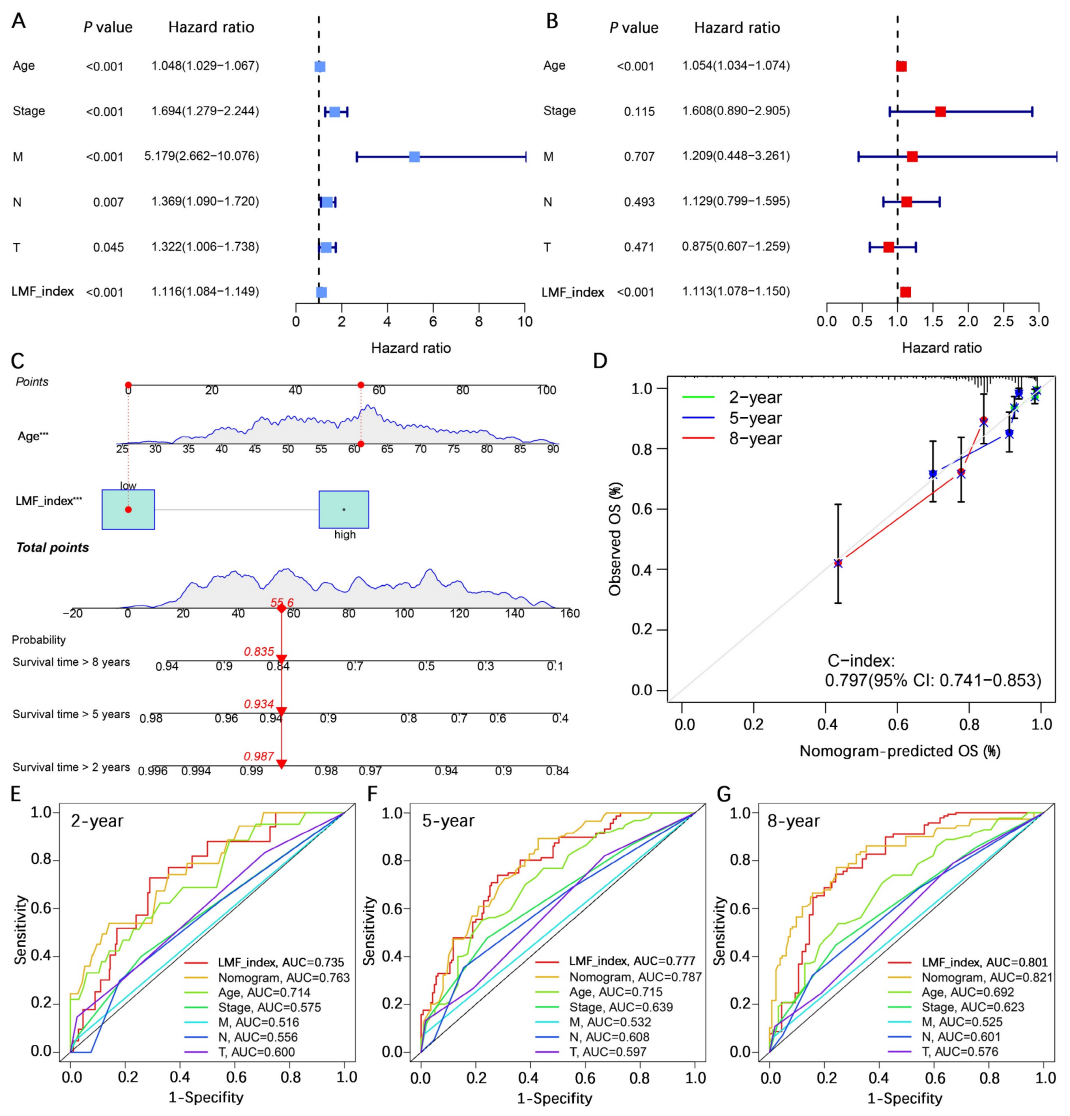
** Independent prognostic analysis of the LMF_index and nomogram construction. (A-B)** Univariate and multivariate Cox regression analyses of clinical-pathological characteristics and LMF_index. **(C)** Construction of a nomogram incorporating LMF_index and clinical-pathological factors. **(D)** Calibration curves for assessing the accuracy of the nomogram in predicting outcomes. **(E-G)** Receiver operating characteristic (ROC) curve analysis of the nomogram, risk score, and clinical-pathological factors for predicting 2-year, 5-year, and 8-year survival.

To assess whether the LMF_index serves as an independent prognostic factor for HR+ breast cancer, univariate and multivariate Cox regression analyses were performed, incorporating key clinical pathological variables including age, stage, T stage, N stage, and M stage. Univariate Cox regression analysis revealed that age (hazard ratio (HR) = 1.048, 95% confidence interval (CI) [1.029-1.067], *P* < 0.001), stage (HR = 1.694, 95% CI [1.279-2.244], *P* < 0.001), M stage (HR = 5.179, 95% CI [2.662-10.076], *P* < 0.001), N stage (HR = 1.369, 95% CI [1.090-1.720], *P* = 0.007), T stage (HR = 1.322, 95% CI [1.006-1.738], *P* = 0.045), and LMF_index (HR = 1.116, 95% CI [1.084-1.149], *P* < 0.001) were significantly associated with patient prognosis (**Figure 5A**). Multivariate Cox regression analysis further confirmed that age (HR = 1.054, 95% CI [1.034-1.074], *P* < 0.001) and LMF_index (HR = 1.113, 95% CI [1.078-1.150], *P* < 0.001) were independent prognostic factors for HR+ breast cancer (**Figure 5B**).

To enhance the accuracy of survival predictions, a nomogram was constructed by integrating age and the LMF_index. Calibration curve analysis demonstrated strong predictive performance, with the nomogram achieving a C-index of 0.797 (95% CI [0.741-0.853]), indicating a high level of concordance between predicted and actual survival outcomes (**Figure 5C-D**). Additionally, multi-variable ROC curve analysis showed that the nomogram exhibited the highest AUC values for 2-year, 5-year, and 8-year survival predictions, with AUCs of 0.763, 0.787, and 0.821, respectively, demonstrating its superior predictive capability compared to individual prognostic factors (**Figure 5E-G**). In summary, the LMF_index was identified as an independent prognostic factor for HR+ breast cancer. Its integration into a nomogram significantly improves survival prediction, providing a valuable tool for individualized prognostic assessment.

### 3.5. Validation of LMF_index in the Shanghai cohort and tissue microarray analysis

**Figure 6:**
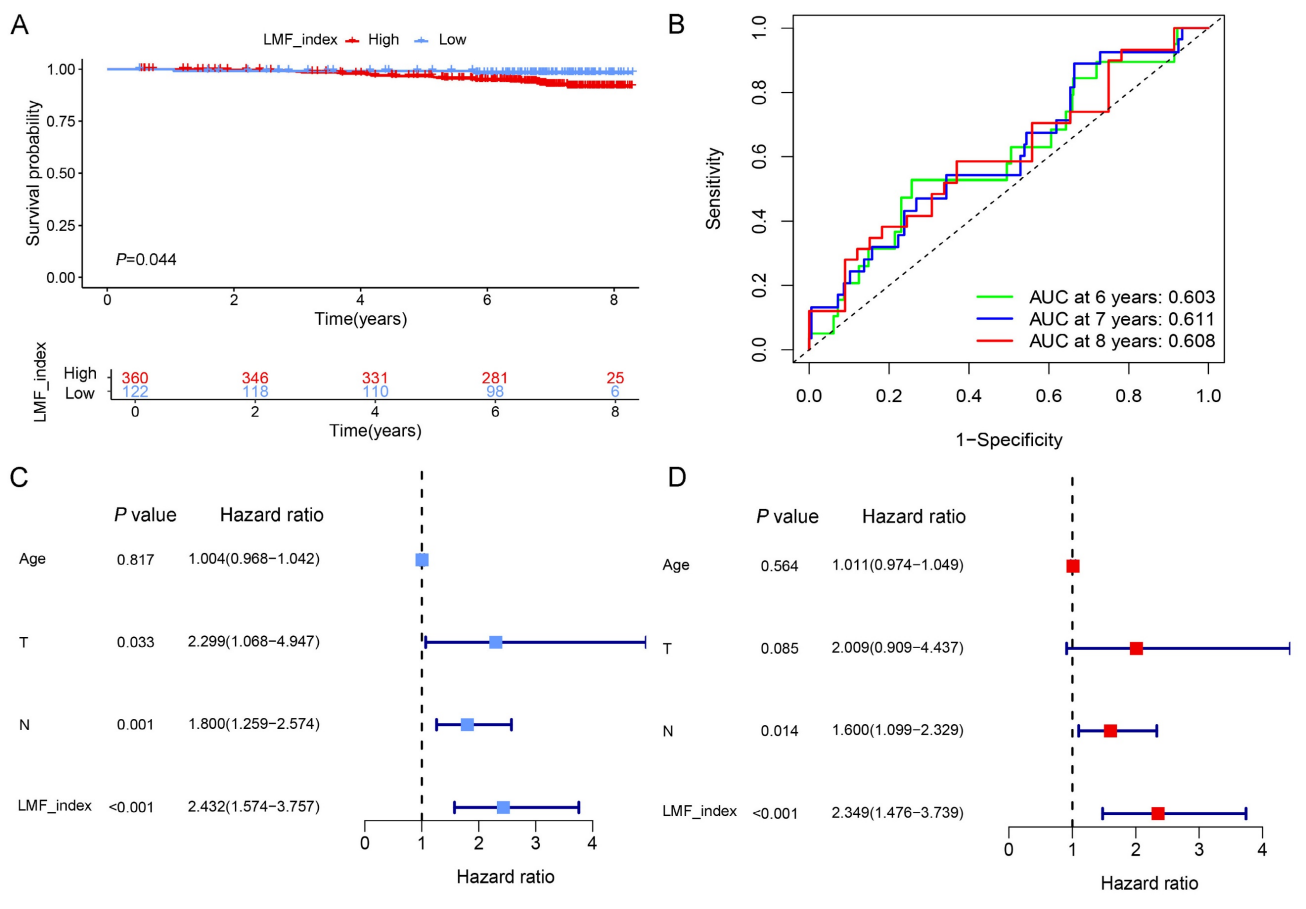
** Validation of the prognostic value of LMF_index in the Shanghai cohort. (A)** Kaplan-Meier survival analysis comparing the LMF_index low and high groups, using the optimal cut-off value. **(B)** Time-dependent receiver operating characteristic (ROC) curves of LMF_index at 6, 7, and 8 years in the Shanghai cohort. **(C-D)** Univariate and multivariate Cox regression analyses of LMF_index combined with clinical and pathological factors in the Shanghai cohort.

To further evaluate the stability and prognostic accuracy of LMF_index, an external validation was performed using the Shanghai cohort, which included 482 HR+ breast cancer patients who received endocrine therapy. Based on the optimal cut-off value, patients were stratified into high LMF_index and low LMF_index groups. Kaplan-Meier survival analysis demonstrated that patients in the high LMF_index group exhibited significantly worse OS compared to those in the low LMF_index group (**Figure 6A**). Moreover, ROC curve analysis revealed that the AUC value for 8-year OS prediction exceeded 0.6, further confirming the predictive reliability of LMF_index in HR+ breast cancer (**Figure 6B**). Additionally, univariate and multivariate Cox regression analyses established LMF_index as an independent prognostic factor for HR+ breast cancer patients (**Figure 6C-D**), highlighting its clinical relevance in prognostic assessment.

**Figure 7:**
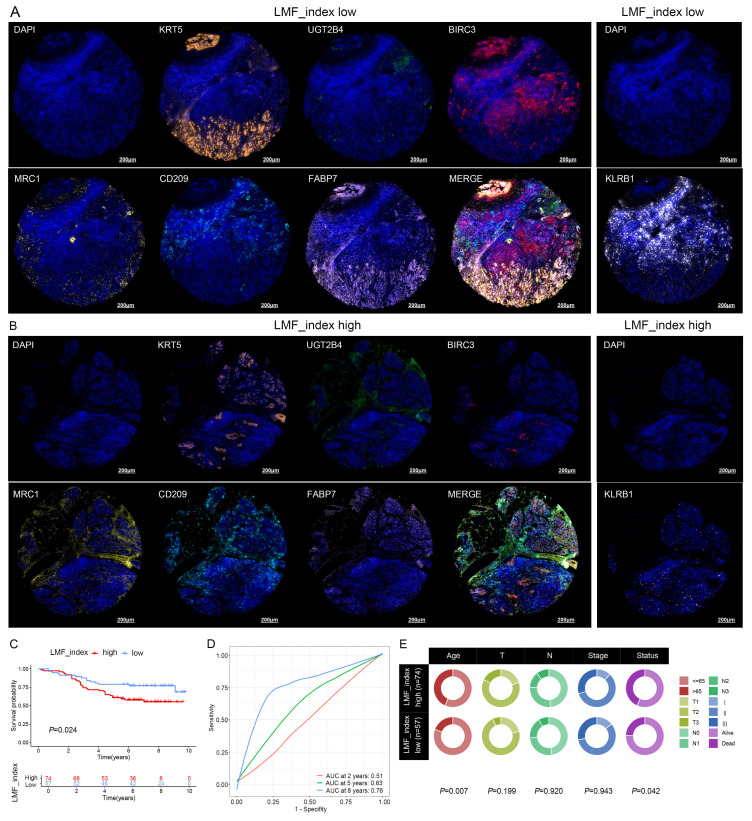
** Prognostic value of LMF_index in breast cancer tissue microarray. (A)** Representative images of KRT5, UGT2B4, BIRC3, MRC1, CD209, FABP7, and KLRB1 based on multiplex immunohistochemistry (mIHC) staining in the LMF_index low group. **(B)** Representative images of KRT5, UGT2B4, BIRC3, MRC1, CD209, FABP7, and KLRB1 based on mIHC staining in the LMF_index high group. **(C)** Kaplan-Meier overall survival (OS) analysis comparing the LMF_index low and high groups, using the optimal cut-off value. **(D)** Time-dependent receiver operating characteristic (ROC) curves of the LMF_index for 2-, 5-, and 8-year OS. **(E)** Circos plot illustrating the distribution of different clinical factors between the LMF_index low and high groups.

To further validate the prognostic significance of LMF_index, mIHC staining was performed on breast cancer tissue microarrays to assess the expression levels of KRT5, UGT2B4, BIRC3, MRC1, CD209, FABP7, and KLRB1. The tissue microarray used in this study comprised samples from 136 breast cancer patients. However, during multiple staining experiments, five tissue spots were found to have detachment issues. As a result, a total of 131 breast cancer patients were included in the subsequent analysis. The LMF_index was calculated based on the proportion of positively stained cells for the seven protein markers. Representative mIHC images demonstrated distinct expression differences between high and low LMF_index groups (**Figure 7A-B**). Kaplan-Meier survival analysis further demonstrated that patients with a low LMF_index had a more favorable prognosis (**Figure 7C**). Furthermore, ROC curve analysis showed that LMF_index achieved an AUC of 0.76 in 8-year OS prediction, underscoring its potential as a robust prognostic biomarker (**Figure 7D**). Notably, LMF_index was significantly associated with patient age and survival status (**Figure 7E**), further supporting its clinical relevance as a prognostic indicator. In conclusion, this study validated the LMF_index in an independent external Shanghai cohort at the transcriptomic level and in a breast cancer tissue microarray at the protein level, confirming its reliability as a prognostic biomarker for HR+ breast cancer.

### 3.6. Analysis of expression levels of LMF_index panel genes

**Figure 8:**
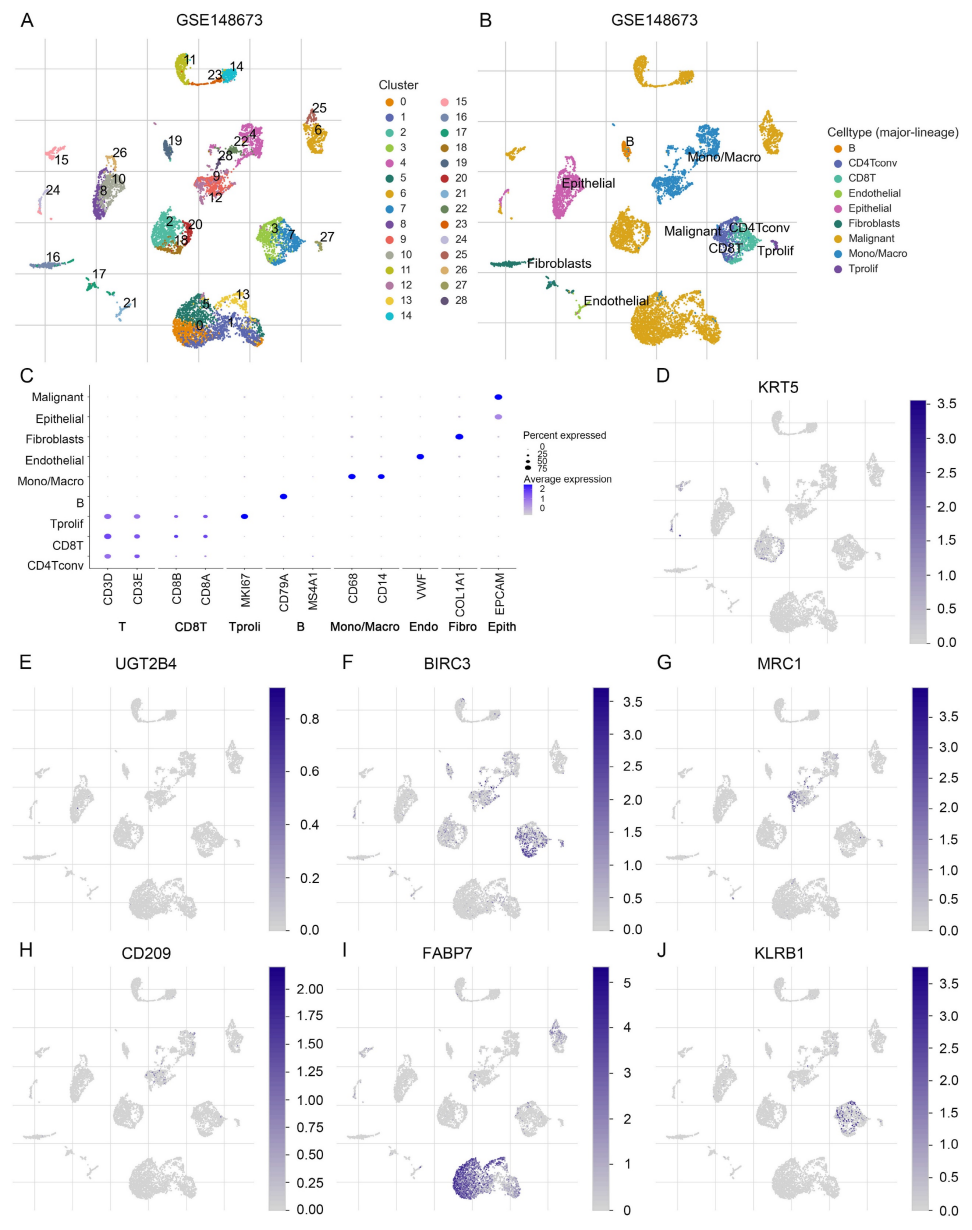
** Single-cell transcriptomic expression levels analysis of LMF_index panel genes. (A)** Dimensionality reduction and clustering of breast cancer tissue using the Uniform Manifold Approximation and Projection (UMAP) method from the TISCH2 database. **(B)** Annotation of 28 clusters into 9 different cell types. **(C)** Dot plot showing the expression levels of marker genes in each cell type. **(D-J)** Analysis of the expression levels of LMF_index panel genes (KRT5, UGT2B4, BIRC3, MRC1, CD209, FABP7, and KLRB1) at the single-cell level in various immune and tumor cell types.

To further investigate the expression characteristics of LMF_index panel genes in breast cancer, we first analyzed their expression levels in normal breast tissue and HR+ breast cancer tissues using the UALCAN database. The results demonstrated that KRT5, UGT2B4, MRC1, CD209, and KLRB1 were significantly downregulated in HR+ breast cancer compared to normal breast tissue, whereas BIRC3 and FABP7 showed no significant differences in expression between the two groups (**Figure S8**). To further elucidate the distribution of these genes in different cell types, we analyzed scRNA-seq data from the GSE148673 dataset. UMAP clustering classified the cells into 28 subpopulations, which were subsequently annotated into 9 major cell types (**Figure 8A-C**). Single-cell expression analysis revealed that KRT5 and FABP7 were highly enriched in tumor cells, UGT2B4 was predominantly expressed in epithelial cells, BIRC3 and KLRB1 were enriched in CD8+ T cells, while MRC1 and CD209 were highly expressed in monocytes/macrophages (**Figure 8D-J and Figure S9**). These findings delineate the distinct expression patterns of LMF_index panel genes in tumor cells and immune cells, providing valuable insights into their potential functional roles in tumor progression and immune regulation.

### 3.7. Immune landscape and immunotherapy sensitivity analysis

Recent studies have demonstrated that cell debris and lipid metabolites released during ferroptosis can enhance tumor-specific immune responses by activating antigen-presenting cells and promoting T cell activation^27^. To further explore the mechanistic basis by which the LMF_index functions as an independent prognostic factor, we investigated the relationships among the LMF_index, ferroptosis, and immune cell infiltration in HR+ breast cancer samples from the TCGA cohort. Correlation analysis revealed that the LMF_index was negatively associated with both the ferroptosis score and immune infiltration score, whereas ferroptosis and immune infiltration scores were positively correlated with each other (**Figure S10A-C**). These findings suggest that the improved prognosis observed in patients with a low LMF_index may be attributed, at least in part, to enhanced ferroptotic activity and more robust immune cell infiltration. ACSL4 has been recognized as a key determinant of ferroptosis sensitivity and has been associated with increased CD8+ T cell infiltration and improved response to ICIs^49^. In contrast, GPX4 is known to suppress ferroptosis by detoxifying lipid peroxides^50^. To further explore their spatial distribution, we employed PanCK staining to distinguish epithelial and stromal regions in tissue microarrays and performed mIHC for PanCK, CD8, ACSL4 and GPX4 (**Figure S10D-E**). The results showed no significant difference in the distribution of ACSL4+ cells between epithelial and stromal regions, whereas GPX4+ cells were significantly more abundant in epithelial compartments (**Figure S10F**). Notably, ACSL4+ cell density was significantly higher in the low LMF_index group compared to the high LMF_index group, while GPX4+ cell distribution did not differ significantly between the two groups (**Figure S10G**). Further correlation analysis revealed a positive association between ACSL4+ cells and CD8+ T cell infiltration, while no such correlation was observed for GPX4+ cells (**Figure S10H-I**). These data suggest that patients in the low LMF_index group exhibit higher ACSL4 expression, indicating increased ferroptosis sensitivity, which may contribute to greater CD8+ T cell infiltration and enhanced anti-tumor immunity.

**Figure 9:**
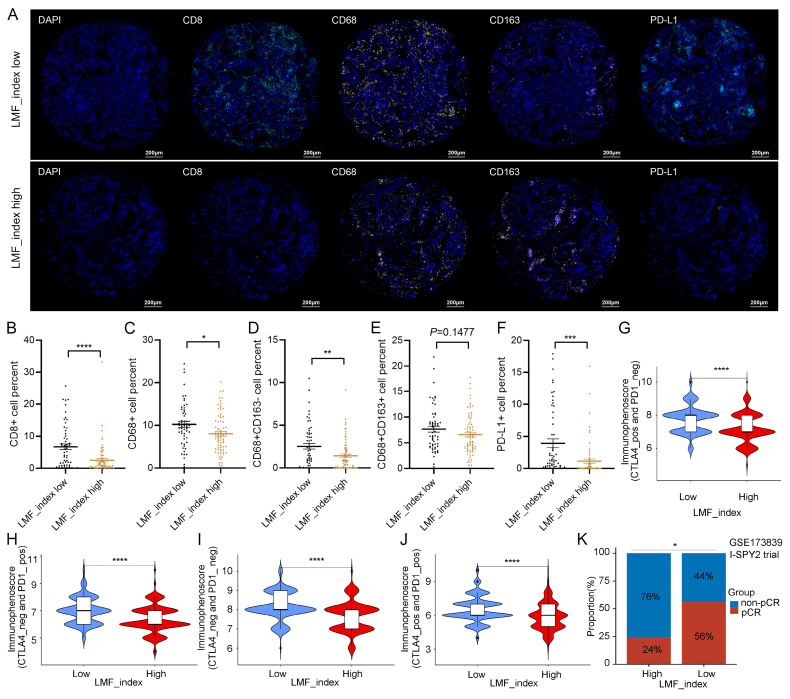
** Immune cell infiltration and immunotherapy sensitivity analysis in different LMF_index groups. (A)** Representative multiplex immunohistochemistry (mIHC) images showing CD8, CD68, CD163, and PD-L1 expression in LMF_index low and high groups. **(B-F)** Quantification of immune cell infiltration, comparing CD8+ T cells, CD68+ (M0-like) macrophages, CD68+CD163- (M1-like) macrophages, CD68+CD163+ (M2-like) macrophages, and PD-L1+ cells between LMF_index low and high groups. **(G-J)** Comparison of immunophenoscore (IPS) between LMF_index low and high groups. **(K)** Comparison of pathological complete response (pCR) rates between LMF_index low and high patients who received neoadjuvant immunotherapy in the GSE173839 dataset. *P < 0.05; **P < 0.01; ***P < 0.001; ****P < 0.0001.

Next, multiple algorithmic analyses revealed a positive correlation between the LMF_index and macrophage infiltration, whereas it was negatively correlated with the infiltration of CD8+ T cells, B cells, and natural killer (NK) cells (**Figure S11A**). Given the association between the LMF_index and immune cell infiltration, we further explored differences in the immune microenvironment between LMF_index high and LMF_index low groups in HR+ breast cancer patients. ESTIMATE analysis revealed that LMF_index low patients exhibited higher stromal scores, immune scores, and total ESTIMATE scores compared to LMF_index high patients, suggesting a more active immune cell infiltration and lower tumor purity in the LMF_index low group (**Figure S11B**). Specifically, LMF_index low patients displayed increased infiltration of naïve B cells, memory B cells, plasma cells, CD8+ T cells, resting memory CD4+ T cells, follicular helper T cells, monocytes, M1 macrophages, resting dendritic cells (DCs), and neutrophils, compared to their LMF_index high counterparts. Conversely, LMF_index high patients exhibited higher infiltration of resting NK cells, M0 macrophages, and M2 macrophages, indicative of a more immunosuppressive TME (**Figure S11C**). Furthermore, ssGSEA analysis confirmed that LMF_index low patients not only demonstrated higher immune cell infiltration but also exhibited enhanced immune-related functional activities, including chemokine receptor signaling, immune checkpoint signaling, human leukocyte antigen (HLA) signaling, and type II interferon response (**Figure S11D**). Additionally, mIHC analysis of the breast cancer tissue microarray confirmed that CD8+ T cell, CD68+ (M0-like) macrophages, and CD68+CD163- (M1-like) macrophages infiltration levels were significantly higher in the LMF_index low group compared to the LMF_index high group. In contrast, no statistically significant difference was observed in CD68+CD163+ (M2-like) macrophages infiltration between the two groups (**Figure 9A-E**).

The efficacy of antitumor therapies in breast cancer is significantly influenced by the TME, which plays a crucial role in determining patient prognosis and treatment response, particularly in immunotherapy^51^, ^52^. Given that the LMF_index reflects the level of immune cell infiltration within the TME, we further investigated its role in predicting immunotherapy response in HR+ breast cancer. We first analyzed its correlation with immune checkpoint expression in HR+ breast cancer. As shown in **Figure S11E**, the LMF_index was correlated with the expression levels of various immune checkpoints in HR+ breast cancer. Subgroup analysis revealed that LMF_index low patients exhibited significantly higher expression of PD-1, PD-L1, and CTLA4 compared to LMF_index high patients (**Figure S11F-H**), suggesting that LMF_index low tumors may be more sensitive to ICIs. Additionally, mIHC analysis of the tissue microarray confirmed that PD-L1 expression levels were significantly higher in the LMF_index low group compared to the LMF_index high group (**Figure 9A, F**). Further validation was performed using the IPS, a biomarker-based scoring system that predicts tumor responsiveness to ICIs, with higher IPS scores indicating a better immunotherapy response^53^. Our findings demonstrated that LMF_index low patients had significantly higher IPS than LMF_index high patients (**Figure 9G-J**), reinforcing the notion that LMF_index low tumors are more likely to benefit from ICIs therapy. Additionally, data from the I-SPY2 clinical trial were analyzed to evaluate real-world neoadjuvant immunotherapy response. HR+ breast cancer patients with LMF_index low exhibited a significantly higher pCR rate following neoadjuvant immunotherapy than LMF_index high patients (*P* < 0.05, **Figure 9K**), further highlighting the potential clinical utility of the LMF_index in guiding immunotherapy selection. In summary, the LMF_index is closely associated with the tumor immune microenvironment, with LMF_index low patients exhibiting higher immune infiltration, increased immune checkpoint expression, and greater responsiveness to immune checkpoint inhibitors, as confirmed by IPS analysis and neoadjuvant immunotherapy outcomes in the I-SPY2 trial.

### 3.8. KLRB1 expression correlates with immune cell infiltration and may serve as a biomarker for immunotherapy response

Single-cell expression analysis of LMF_index panel genes revealed that BIRC3 and KLRB1 were predominantly enriched in T cells. Differential expression analysis between normal breast tissue and HR+ breast cancer tissue showed significantly lower KLRB1 expression in tumor tissues, whereas BIRC3 expression exhibited no significant difference. Furthermore, univariate Cox regression analysis identified KLRB1 as a protective factor (HR < 1, *P* < 0.05), with its expression negatively correlated with LMF_index (R = -0.432, *P* < 0.001) (**Figure S12A**). Paired breast cancer tissue analysis confirmed that KLRB1 expression was significantly lower in tumors compared to adjacent normal tissues (**Figure S12B**). The mIHC results showed that patients with high KLRB1 expression had better prognosis and higher PD-L1 expression levels (**Table 1**). Survival analysis using the Kaplan-Meier Plotter database further demonstrated that higher KLRB1 expression was associated with significantly better OS and RFS in HR+ breast cancer patients (**Figure S12C**).

**Figure 10:**
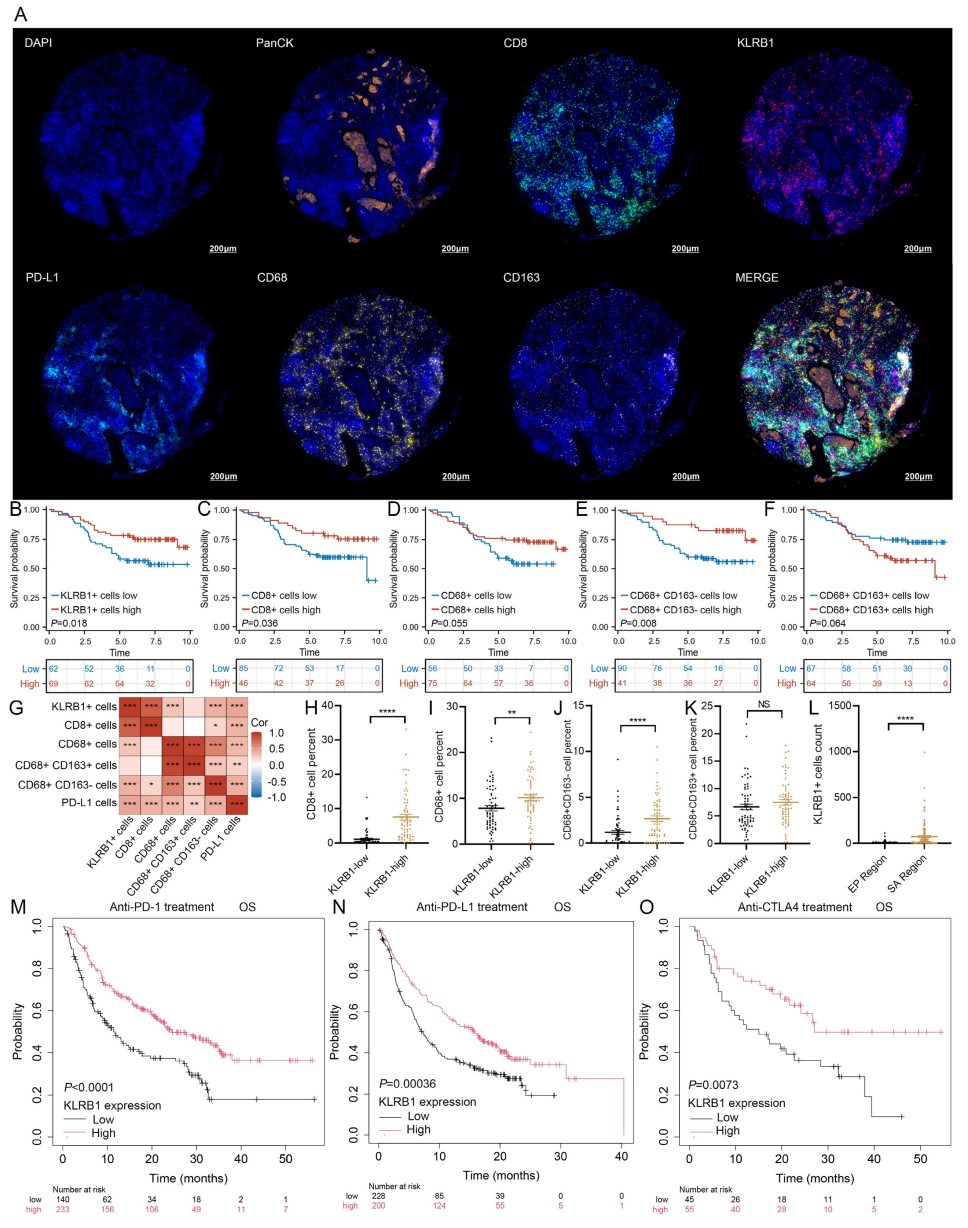
** KLRB1 expression and immune cell infiltration analysis in breast cancer tissue microarray. (A)** Representative multiplex immunohistochemistry (mIHC) images showing PanCK, CD8, KLRB1, PD-L1, CD68, and CD163 expression in the KLRB1 high group. **(B-F)** Kaplan-Meier overall survival (OS) analysis comparing the infiltration levels of KLRB1+ cells, CD8+ T cells, CD68+ (M0-like) macrophages, CD68+CD163- (M1-like) macrophages, and CD68+CD163+ (M2-like) macrophages. **(G)** Correlation heatmap analysis of the percentages of KLRB1+ cells, CD8+ T cells, CD68+ (M0-like) macrophages, CD68+CD163- (M1-like) macrophages, and CD68+CD163+ (M2-like) macrophages, and PD-L1+ cells. **(H-K)** Infiltration level differences in CD8+ T cells, CD68+ (M0-like) macrophages, CD68+CD163- (M1-like) macrophages, and CD68+CD163+ (M2-like) macrophages between KLRB1+ cells high and low breast cancer patients. **(L)** Differential analysis of KLRB1+ cells between stromal and epithelial regions. **(M-O)** Kaplan-Meier overall survival analysis based on KLRB1 expression levels in patients receiving PD-1 inhibitors, PD-L1 inhibitors, and CTLA4 inhibitors.

To investigate the role of KLRB1 in the tumor immune microenvironment, we examined its correlation with immune cell infiltration. Heatmap analysis indicated that KLRB1 expression was positively correlated with multiple immune cell populations, including T cells, B cells, and macrophages (**Figure S12D**). H&E staining further validated that breast cancer tissues with high KLRB1 expression exhibited increased lymphocyte infiltration (**Figure S12E**). To further assess the relationship between KLRB1 expression and immune cell infiltration, mIHC staining was performed on breast cancer tissue microarrays, targeting PanCK, KLRB1, CD8, CD68, CD163, and PD-L1. Representative images in **Figure 10A and Figure S12F** demonstrated distinct immune infiltration patterns between patients with low and high KLRB1 expression. Protein expression levels were quantified based on the proportion of marker-positive cells, and survival analysis was conducted accordingly. The results indicated that patients with a higher proportion of KLRB1-positive cells exhibited significantly better prognosis (**Figure 10B**). Similarly, higher proportions of CD8+ T cells (**Figure 10C**) and CD68+CD163- macrophages (**Figure 10E**) were associated with improved survival outcomes. In contrast, CD68+ cells and CD68+CD163+ macrophages showed no significant correlation with prognosis (**Figures 10D, F**). Correlation analysis further revealed that KLRB1+ cells were positively associated with CD8+ T cells, CD68+ macrophages, CD68+CD163- macrophages, and PD-L1+ cells (**Figure 10G**). Subgroup analysis demonstrated that patients with high KLRB1 expression exhibited significantly higher infiltration levels of CD8+ T cells, M0 macrophages, and M1 macrophages compared to those with low KLRB1 expression (**Figure 10H-J**). However, M2 macrophage infiltration levels did not differ significantly between the two groups (**Figure 10K**). Furthermore, spatial analysis based on PanCK expression revealed that KLRB1 expression was significantly higher in the stromal compartment than in the epithelial compartment, suggesting its predominant presence in immune cells (**Figure 10L**). Notably, Kaplan-Meier survival analysis of immunotherapy-treated patients from the Kaplan-Meier Plotter database demonstrated that high KLRB1 expression was associated with prolonged OS in patients receiving PD-1, PD-L1, or CTLA-4 blockade therapy (**Figure 10M-O**). These findings highlight KLRB1 as a key regulator of the immune microenvironment, demonstrating its strong correlation with CD8+ T cell and M1 macrophage infiltration. Moreover, KLRB1 may serve as a potential prognostic biomarker and predictor of immunotherapy response, offering novel insights for optimizing immunotherapeutic strategies.

## 4. Discussion

In the present study, we identified distinct LMF-related molecular clusters in HR+ breast cancer, providing new insights into the prognostic and immunological diversity. Our findings reveal that patients in cluster 1, characterized by higher TMB, more active proliferative signaling (PI3K/AKT/mTOR), and elevated Ki-67 expression, exhibited more aggressive tumor features and relatively poorer clinical outcomes. In contrast, cluster 2 demonstrated greater immune cell infiltration, higher expression of immune checkpoints (PD-1, PD-L1, and CTLA-4), and a more favorable prognosis. These results are consistent with previous studies suggesting that high TMB and elevated proliferation markers are associated with more aggressive tumor behavior in HR+ breast cancer^54^, ^55^, whereas robust tumor-infiltrating lymphocytes (TILs) and higher immune checkpoint expression often correlate with improved outcomes and responsiveness to immunotherapy in various solid tumors^56^.

Building upon the molecular clustering analysis, we developed the LMF_index and conducted comprehensive validation across two internal and four external validation cohorts. Further analysis demonstrated that the LMF_index effectively stratifies HR+ breast cancer patients into LMF_index low and LMF_index high subgroups. Notably, patients in the LMF_index low group exhibited significantly better OS than those in the LMF_index high group. This trend was consistently observed across various clinicopathological subgroups, further reinforcing the robustness of the LMF_index as a prognostic classifier. Moreover, multivariate Cox regression analysis confirmed that the LMF_index is an independent prognostic factor for HR+ breast cancer, highlighting its potential clinical utility. Compared to previously established lipid metabolism-related and ferroptosis-related indices^28^-^31^, the LMF_index integrates two tightly linked biological processes, demonstrating superior predictive accuracy and stability in prognosis assessment. ROC curve analysis showed that the AUC values for 2-, 5-, and 8-year survival predictions were consistently higher for the LMF_index than for other indices^28^-^31^, indicating its enhanced long-term prognostic capability.

Furthermore, C-index analysis confirmed that the LMF_index outperforms existing lipid metabolism and ferroptosis-related indices in predicting HR+ breast cancer prognosis^28^-^31^, highlighting its potential for clinical application. Notably, in contrast to previous lipid metabolism or ferroptosis indices, our LMF_index was further validated in our independent Shanghai cohort and breast cancer tissue microarray, providing additional evidence of its reliability and clinical applicability. These results further support the LMF_index as a robust prognostic biomarker with the potential for real-world clinical implementation in HR+ breast cancer.

The LMF_index panel consists of seven key genes: KRT5, UGT2B4, BIRC3, MRC1, CD209, FABP7, and KLRB1. These genes were identified through a rigorous selection process, including LASSO regression and multivariate Cox analysis, to ensure their prognostic significance and functional relevance in HR+ breast cancer. Keratin 5 (KRT5) is a member of the keratin family, highly expressed in basal-like breast cancer and commonly used as a marker for basal-like subtype identification^57^. KRT5+ cells exhibit strong cancer stem cell (CSC) properties, which have been associated with endocrine therapy resistance and poor prognosis in ER+ breast cancer^58^, ^59^. Additionally, studies suggest that KRT5-β-catenin interaction plays a critical role in maintaining the malignant phenotype of breast cancer cells, and targeting this pathway may serve as a novel therapeutic strategy^58^. UGT2B4, a member of the UDP-glucuronosyltransferase (UGT) family, plays a pivotal role in hepatic metabolism and drug biotransformation by catalyzing the glucuronidation of various endogenous and exogenous substrates^60^. Emerging evidence indicates that UGT2B4 contributes to doxorubicin resistance in breast cancer cells, potentially through somatic mutations that impair its normal enzymatic function or alter its expression^61^, ^62^. However, the underlying molecular mechanisms driving this resistance remain incompletely understood. Beyond its role in drug metabolism, UGT2B4 has been identified as a downstream target of peroxisome proliferator-activated receptor alpha (PPARα), implicating it in lipid and cholesterol metabolism^63^. As such, UGT2B4 is increasingly recognized as a key regulator of metabolic homeostasis, often referred to as a “lipid controller”^64^. Recent transcriptomic analyses have further expanded the biological significance of UGT2B4 in cancer. A study by Wang *et al.* identified UGT2B4 as a characteristic gene of ferroptosis-related molecular subtypes^65^. This observation is consistent with our current findings and supports the notion that UGT2B4 may serve a dual function—participating in both lipid metabolic reprogramming and the regulation of ferroptosis—thereby contributing to tumor progression and therapeutic response. BIRC3, a member of the inhibitor of apoptosis protein (IAP) family, primarily regulates cell survival by modulating the NF-κB signaling pathway to inhibit apoptosis^66^. In breast cancer, BIRC3 upregulation has been associated with enhanced tumor cell survival and doxorubicin resistance^67^. Notably, a study by Feng *et al.* identified BIRC3 as a protective factor in breast cancer patients^68^, which is consistent with the findings of this study. MRC1 is primarily expressed in M2 macrophages and plays a crucial role in shaping an immunosuppressive TME. In colorectal cancer liver metastases, highly metabolically active MRC1+CCL18+ M2 macrophages have been identified and are associated with poor response to neoadjuvant chemotherapy^69^. In breast cancer, TAp73 expression is negatively correlated with the accumulation of pro-tumorigenic MRC1+ macrophages in patient samples. Mechanistically, TAp73 regulates the accumulation and phenotype of MRC1+ macrophages by suppressing the NF-κB signaling pathway^70^. High MRC1 expression may indicate the enrichment of M2 macrophages within the TME, suggesting an enhanced immunosuppressive state, which could influence patient response to immunotherapy. CD209 is primarily expressed in DCs and plays a key role in antigen presentation and immune regulation^71^. In breast cancer, CD209 may influence the efficacy of ICIs by modulating DCs function. Fatty acid-binding proteins (FABPs) constitute a family of intracellular lipid chaperones that facilitate the trafficking and utilization of long-chain fatty acids within cells. Among them, FABP7 has garnered attention for its multifaceted role in lipid metabolism and cancer biology^72^, ^73^. FABP7 binds oleic acid (OA) to form FABP7-OA complex, which promotes the formation of nuclear lipid droplets, thereby contributing to lipid storage and signaling regulation^74^. Beyond fatty acid transport, FABP7 has also been recognized as a novel sterol transfer protein that mediates cholesterol efflux from lysosomes to the cytoplasm, playing a key role in maintaining intracellular cholesterol homeostasis^75^. In the context of cancer, FABP7 has been implicated in metabolic reprogramming of breast cancer cells, where it promotes glycolysis and lipid droplet accumulation, supporting tumor growth and survival^76^. Interestingly, FABP7 expression is frequently downregulated in breast cancer tissues, and it has been proposed as a potential biomarker for predicting response to neoadjuvant chemotherapy^77^. Moreover, recent evidence suggests a more nuanced role for FABP7 in the tumor immune microenvironment. Specifically, FABP7 upregulation in immunotherapy-resistant tumors has been shown to confer protection against ferroptosis by modulating lipid composition and mitochondrial function^78^. KLRB1 encodes CD161, which is predominantly expressed on NK cells and CD8+ T cells, playing a crucial role in antitumor immunity^79^. In this study, we found that high KLRB1 expression was associated with increased infiltration of CD8+ T cells and M1 macrophages, and patients with elevated KLRB1 levels exhibited a better response to immunotherapy. These findings suggest that KLRB1 may serve as a potential predictive biomarker for immunotherapy efficacy in HR+ breast cancer. The LMF_index panel genes encompass key biological processes, including tumor stemness, metabolic regulation, apoptosis and survival, and immune modulation within the TME. Their collective function may influence HR+ breast cancer progression and response to immunotherapy.

The LMF_index developed in this study effectively stratifies HR+ breast cancer patients and is closely associated with TME characteristics. HR+ breast cancer is typically characterized by an immune-cold TME, with low immune cell infiltration and reduced immune activation^16^. However, emerging studies suggest that the TME of HR+ breast cancer exhibits plasticity, and not all patients present with the same immune profile. For instance, a subset of HR+ breast cancer patients demonstrates higher infiltration of CD8+ T cells and M1 macrophages, contributing to a more robust anti-tumor immune response^80^, ^81^. In this study, ESTIMATE analysis revealed that patients in the LMF_index low group exhibited significantly higher immune and stromal scores than those in the LMF_index high group, indicating a TME enriched with immune-active components in the former, while the latter exhibited higher tumor purity. Furthermore, LMF_index low patients displayed increased levels of CD8+ T cells, M1 macrophages, plasma cells, and follicular helper T cells, whereas LMF_index high patients were enriched in M2 macrophages and resting NK cells. These findings align with the classical TME paradigm, where M1 macrophages and CD8+ T cells are associated with an enhanced anti-tumor immune response, whereas M2 macrophages contribute to tumor progression and immune suppression^82^.

ICIs have demonstrated significant efficacy in TNBC; however, their clinical progress in HR+ breast cancer has been relatively slow^16^. The KEYNOTE-756 and CheckMate 7FL trials have shown that ICIs combined with neoadjuvant chemotherapy can improve the pCR rate in HR+ breast cancer patients^18^, ^19^. Nevertheless, identifying HR+ breast cancer patients who are most likely to benefit from immunotherapy remains a critical clinical challenge. In this study, we assessed the ability of the LMF_index to predict ICIs response using the IPS. The results showed that patients with a low LMF_index exhibited significantly higher IPS scores than those with a high LMF_index, suggesting that the LMF_index low group may be more responsive to PD-1/PD-L1 inhibitors. Furthermore, analysis of the I-SPY2 clinical trial dataset revealed that patients in the LMF_index low group had a significantly higher pCR rate following neoadjuvant immunotherapy compared to those in the LMF_index high group, further validating the predictive value of the LMF_index for immunotherapy outcomes. Additionally, mIHC analysis demonstrated that KLRB1 expression was higher in HR+ breast cancer patients with a low LMF_index, and patients with high KLRB1 expression also exhibited elevated PD-L1 levels. These findings align with previous studies indicating that patients with high PD-L1 expression have a better response to immunotherapy^18^, ^19^. Therefore, the LMF_index holds potential as a biomarker for immunotherapy selection in HR+ breast cancer, aiding in patient stratification and enhancing the precision of immunotherapy strategies.

This study has several strengths. First, we identified two distinct molecular subtypes of HR+ breast cancer, providing new insights into tumor heterogeneity and immune microenvironment characteristics. Second, we developed the LMF_index, a robust prognostic biomarker, which was validated across multiple independent cohorts, including our Shanghai cohort, demonstrating superior prognostic performance compared to existing indices. Additionally, protein-level validation using mIHC on tissue microarrays further strengthens the reliability and applicability of this biomarker. Third, our findings indicate that patients with a low LMF_index exhibit a more active immune microenvironment and may derive greater benefit from ICIs, underscoring its potential role in guiding immunotherapy strategies. Furthermore, we identified KLRB1 as a key immune-related gene, positively correlated with CD8+ T cell and M1 macrophage infiltration, and associated with improved immunotherapy response, making it a promising biomarker for patient stratification in clinical practice.

Despite these strengths, several limitations should be acknowledged. First, although the LMF_index was validated across multiple cohorts and at the protein level using tissue microarrays, its clinical utility requires further confirmation through prospective clinical trials and larger datasets. Second, while this study demonstrates that the LMF_index can predict immunotherapy efficacy, additional prospective clinical studies are needed to validate its role in guiding treatment decisions. Third, although we identified strong associations between the LMF_index, immune landscape features, and patient prognosis, further functional validation is warranted. Specifically, *in vitro* and *in vivo* experiments are needed to elucidate the mechanistic roles of key LMF_index genes—such as BIRC3, FABP7, and UGT2B4—in regulating lipid metabolism, ferroptosis sensitivity, and immune evasion. Fourth, although bulk transcriptomic analysis provides valuable insights, future studies integrating single-cell metabolomics with single-cell transcriptomic data are warranted to further validate and refine the biological relevance of the LMF_index in HR+ breast cancer at a higher cellular resolution. Finally, this study primarily focused on HR+ breast cancer; future research should investigate whether this integrative index can be applied to other breast cancer subtypes or malignancies to expand its clinical relevance.

## 5. Conclusion

In conclusion, this study identified distinct LMF-related molecular clusters in HR+ breast cancer, each exhibiting unique prognostic and immune characteristics. Furthermore, the LMF_index represents a promising prognostic and predictive biomarker for HR+ breast cancer, offering potential guidance for immunotherapy stratification. However, further validation through functional studies and prospective clinical trials is essential to establish its broader clinical applicability.

## Supplementary Material

Supplementary figures.

Supplementary tables.

## Figures and Tables

**Table 1:** Association between KLRB1 expression and clinicopathological characteristics in breast cancer tissue microarray.

Characteristics	KLRB1 low (n=66)	KLRB1 high (n=65)	*P* value	Statistic	Method
Age, median (IQR)	60 (50.25, 71)	54 (49, 67)	0.111	-	Wilcoxon
T, n (%)			0.565	1.1425	Chisq test
T1	15 (11.5%)	12 (9.2%)			
T2	41 (31.3%)	46 (35.1%)			
T3	10 (7.6%)	7 (5.3%)			
N, n (%)			0.186	4.8124	Chisq test
N0	33 (25.2%)	30 (22.9%)			
N1	21 (16%)	14 (10.7%)			
N2	6 (4.6%)	14 (10.7%)			
N3	6 (4.6%)	7 (5.3%)			
Stage, n (%)			0.276	2.5777	Chisq test
I	9 (6.9%)	8(6.1%)			
II	42 (32.1%)	34 (26%)			
III	15 (11.5%)	23 (17.6%)			
Status, n (%)			0.033	4.5466	Chisq test
Dead	29 (22.1%)	17 (13%)			
Alive	37 (28.2%)	48 (36.6%)			
PD-L1+ cells percent, median (IQR)	0.075 (0.02, 0.652)	1.25 (0.15, 5.68)	< 0.001	-	Wilcoxon

## Abbreviations

AIs: aromatase inhibitors
AUC: area under the curve
CSC: cancer stem cell
C-index: concordance index
CI: confidence interval
CDF: cumulative distribution function
CDK4/6: cyclin-dependent kinase 4/6
DCs: dendritic cells
DEGs: differential expression genes
DFS: disease-free survival
mDNAsi: DNA stemness index
ER: estrogen receptor
ERα: estrogen receptor alpha
GO: gene ontology
IC50: half-maximal inhibitory concentration
HR: hazard ratio
H&E: hematoxylin and eosin
HR+: hormone receptor-positive
HER2: human epidermal growth factor receptor 2
HLA: human leukocyte antigen
ICIs: immune checkpoint inhibitors
IPS: immunophenoscores
KEGG: kyoto encyclopedia of genes and genomes
LASSO: least absolute shrinkage and selection operator
LMF: lipid metabolism and ferroptosis
NK: natural killer
PFS: progression-free survival
OA: oleic acid
OS: overall survival
pCR: pathological complete response
PCA: principal component analysis
PR: progesterone receptor
ROC: receiver operating characteristic
RFS: recurrence-free survival
RCB: residual cancer burden
mRNAsi: RNA stemness index
scRNA-seq: single-cell RNA sequencing
SERMs: selective estrogen receptor modulators
SERDs: selective estrogen receptor degraders
ssGSEA: single-sample gene set enrichment analysis
t-SNE: t-distributed stochastic neighbor embedding
TCGA-BRCA: the cancer genome atlas-breast invasive carcinoma
TCIA: the cancer immunome atlas
TNBC: triple-negative breast cancer
TISCH2: Tumor Immune Single-cell Hub 2
TME: tumor microenvironment
TMB: tumor mutational burden
UALCAN: University of Alabama at Birmingham Cancer data analysis portal
