# Supplementary material for: Multi-cohort validation of a lipid metabolism and ferroptosis-associated index for prognosis and immunotherapy response prediction in hormone receptor-positive breast cancer
Source: Int J Biol Sci. 2025 Jun 9;21(9):3968–92. doi: 10.7150/ijbs.113213 (PMC12223777; doi:10.7150/ijbs.113213)
Supplement: Supplementary file 1 — Supplementary figures. [file ijbsv21p3968s1.pdf]

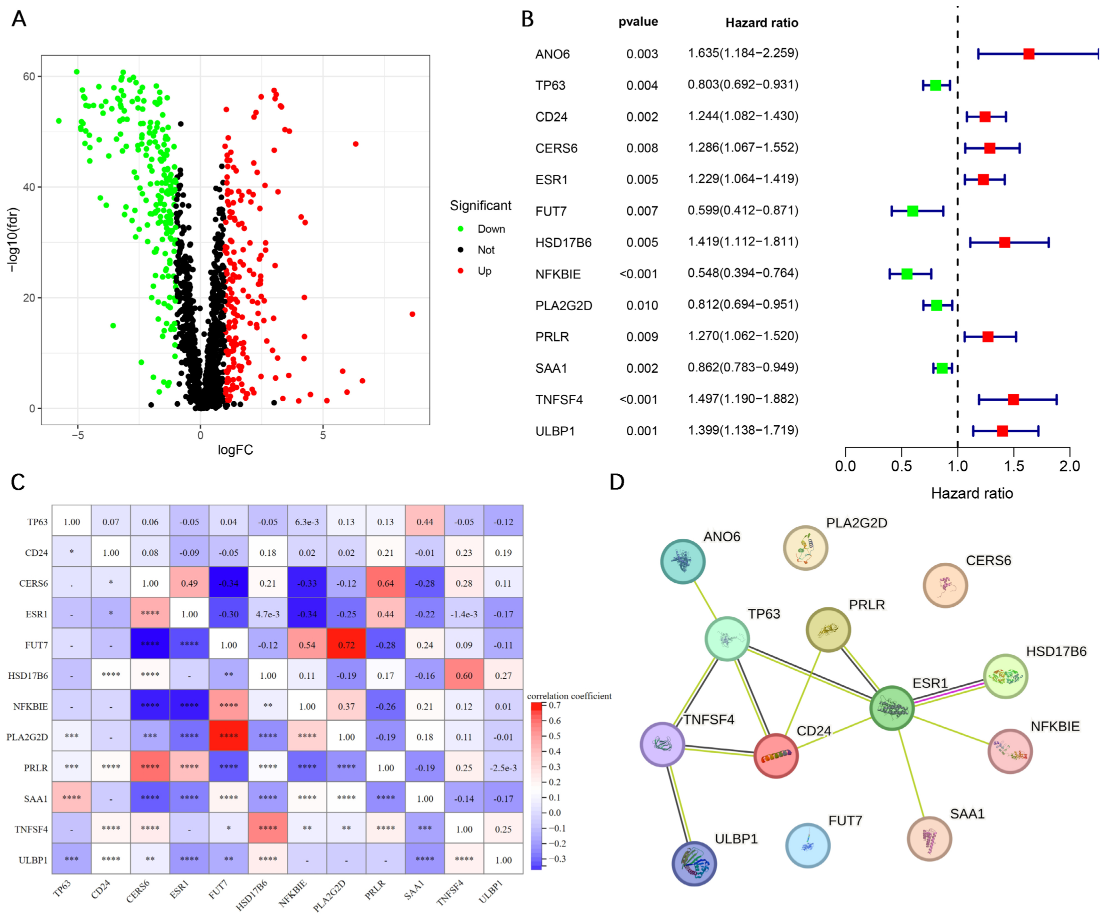

Figure S1. Expression and prognostic analysis of LMF-related genes in HR+ breast cancer.

(A) Volcano plot illustrating the differential expression analysis of LMF-related genes. **(B)** Forest plot of univariate Cox regression analysis for differentially expressed LMF-related genes. **(C)** Heatmap showing the Spearman correlation of expression of prognostic LMF-related genes in HR+ breast cancer. **(D)** Protein-protein interaction network of prognostic LMF-related genes in HR+ breast cancer.

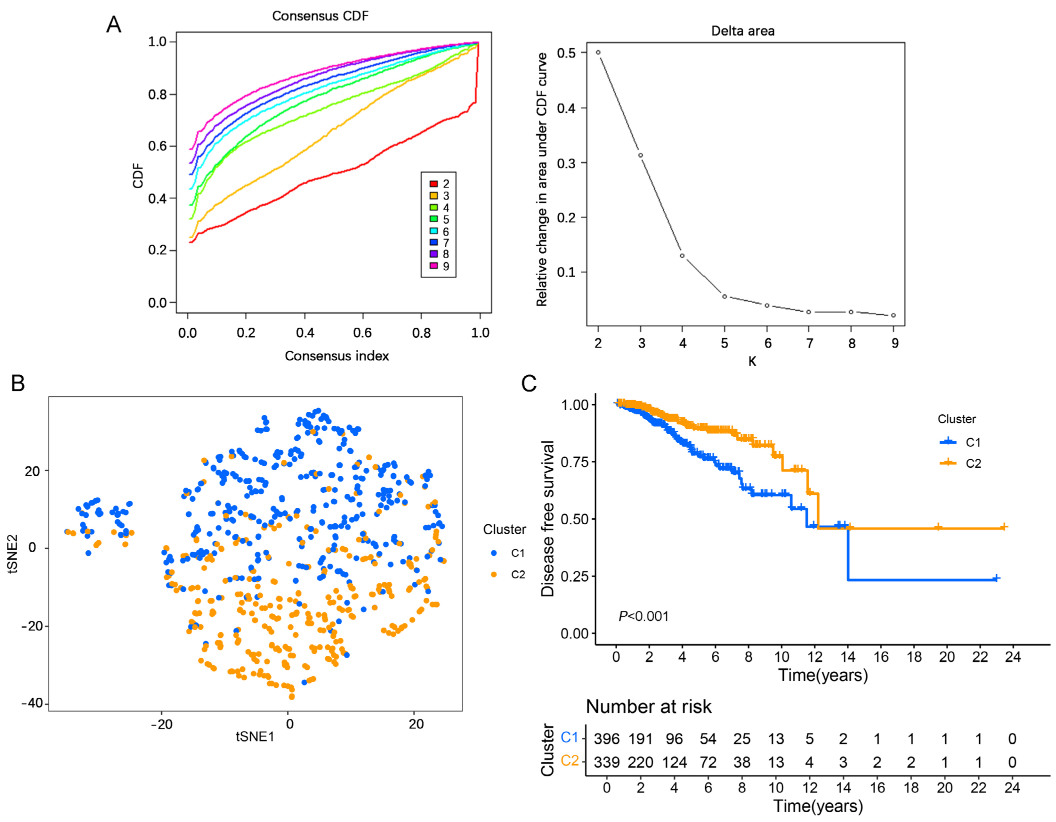

Figure S2. Molecular clusters of LMF-related genes in HR+ breast cancer, related to Figure 2.

(A-B) The consensus cumulative distribution function (CDF) curve and Delta area plot of unsupervised clustering analysis. **(C)** t-Distributed Stochastic Neighbor Embedding (t-SNE) plot illustrating the distribution of the two molecular clusters. **(D)** Kaplan-Meier survival curve comparing disease-free survival (DFS) between the two molecular clusters.

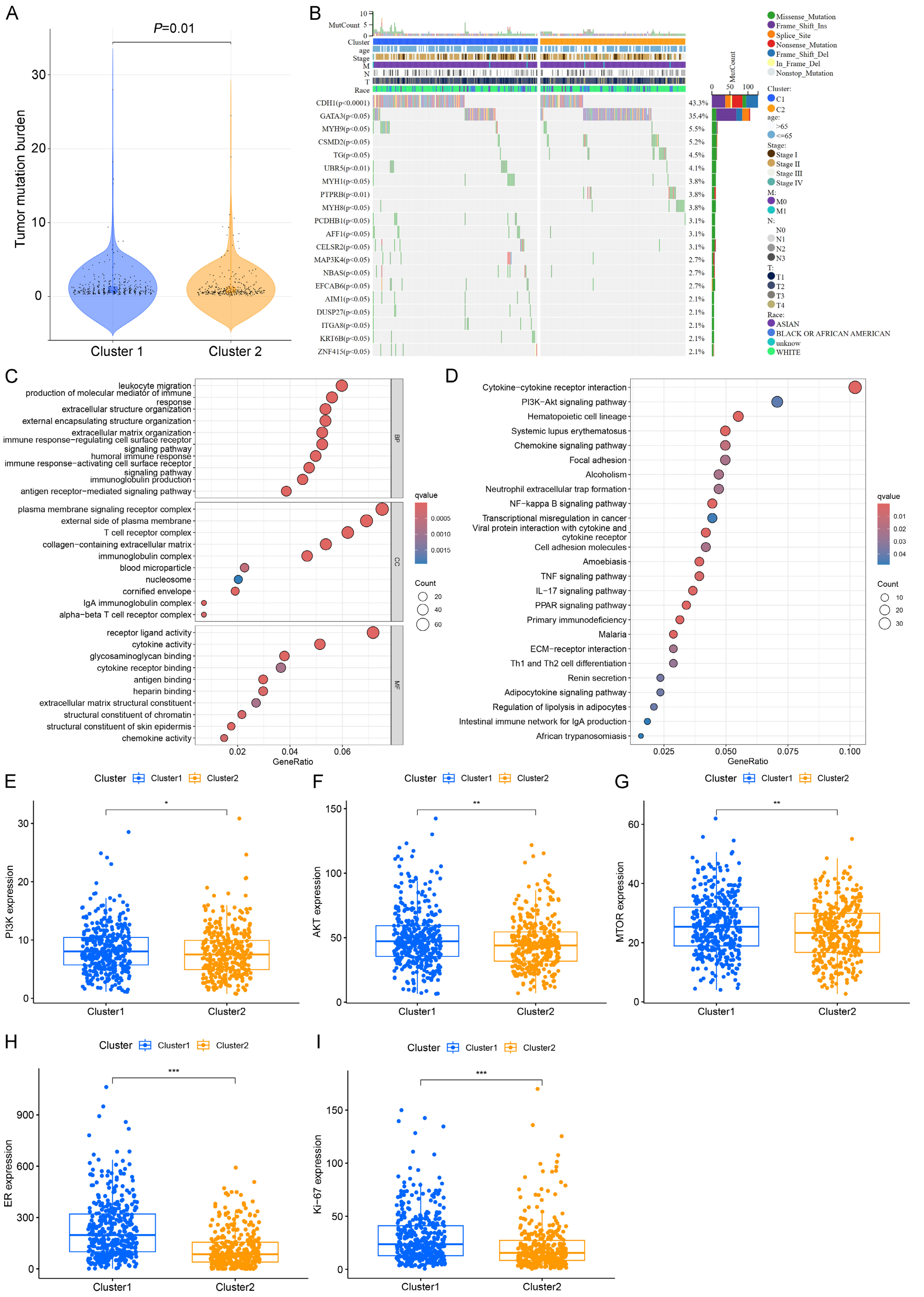

Figure S3. Biological functional analysis between different molecular clusters in HR+ breast cancer, related to Figure 3.

(A) Differential analysis of tumor mutational burden between the two molecular clusters in HR+ breast cancer. **(B)** Waterfall plot illustrating the differences in gene mutations between the two molecular clusters. **(C-D)** Gene Ontology (GO) functional enrichment and Kyoto Encyclopedia of Genes and

Genomes (KEGG) pathway analyses of differentially expressed genes (DEGs) between the two molecular clusters. **(E-I)** Comparative analysis of the expression levels of PI3K, AKT, MTOR, ER, and Ki-67 between the two molecular clusters.

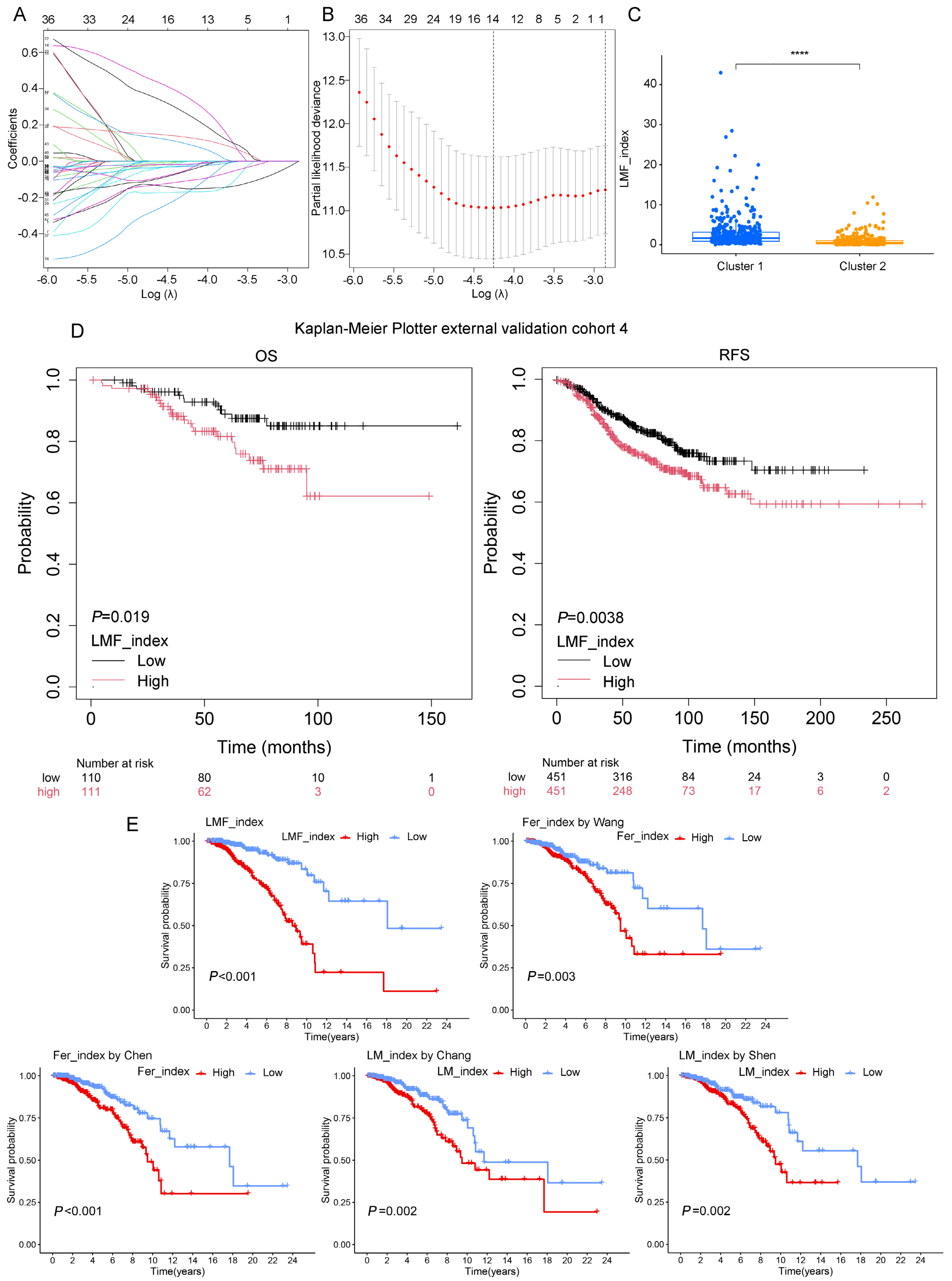

Figure S4. Construction and Validation of the LMF_index and comparison with existing indices, related to Figure 4.

(A) Variable selection and regression coefficient paths from the least absolute shrinkage and selection operator (LASSO) regression analysis. **(B)** Optimal λ value determined by 10-fold cross-validation during LASSO regression, illustrating the selection of the most predictive λ value for LASSO regression. **(C)** Comparison of LMF_index across different molecular clusters. **(D)** Kaplan-Meier overall survival (OS) analysis and recurrence-free survival (RFS) analysis of LMF_index in external validation cohort 4. **(E)** Kaplan-Meier overall survival analysis based on LMF_index and other indices. ****$P < 0.0001$.

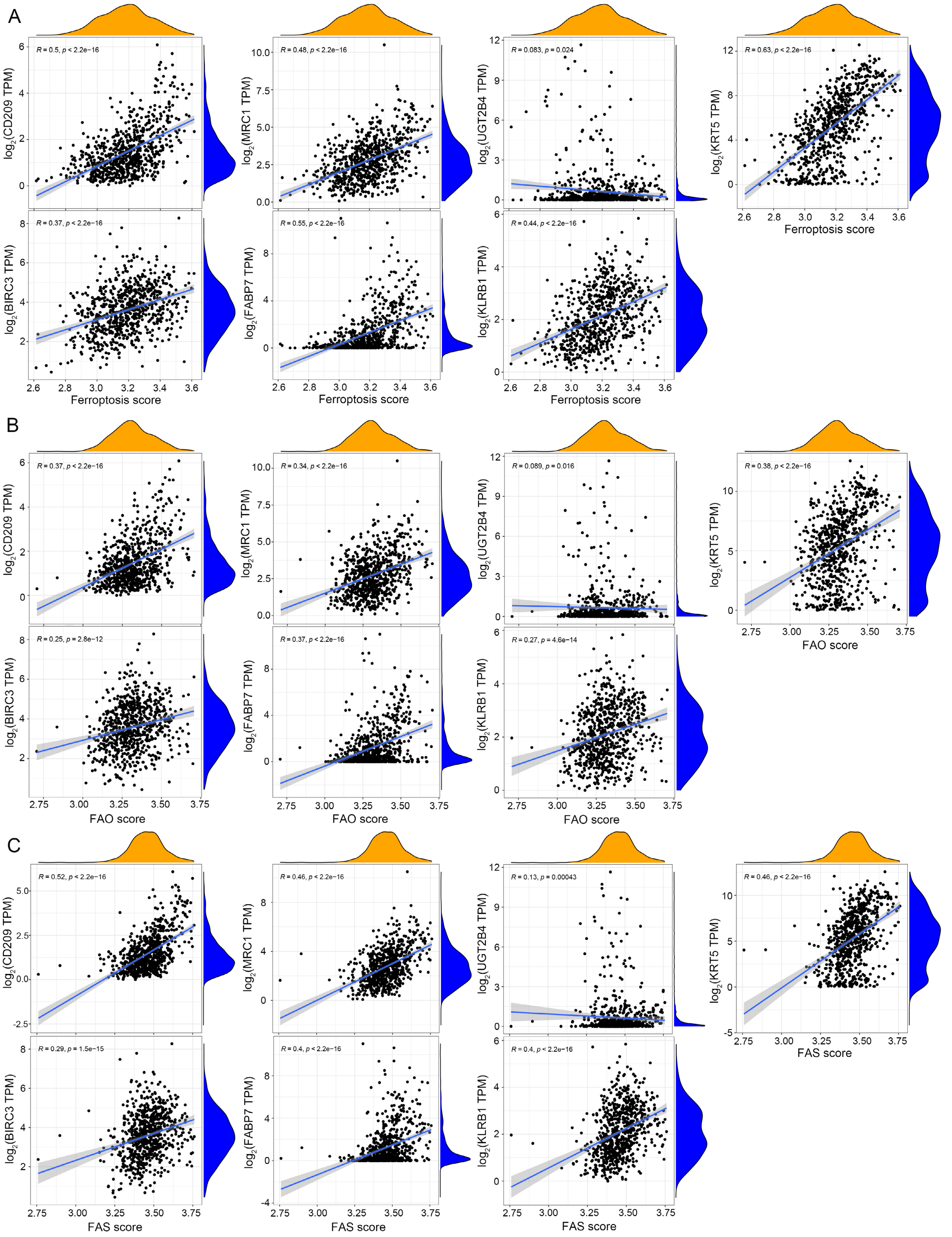

Figure S5. Correlation analysis between LMF_index panel genes and pathway activity scores of ferroptosis, fatty acid oxidation, and fatty acid synthesis in HR+ breast cancer.

(A) Spearman correlation analysis between CD209, MRC1, UGT2B4, KRT5, BIRC3, FABP7, and KLRB1 and ferroptosis scores derived from the ssGSEA algorithm. **(B)** Spearman correlation analysis between CD209, MRC1, UGT2B4, KRT5, BIRC3, FABP7, and KLRB1 and fatty acid oxidation (FAO) pathway scores. **(C)** Spearman correlation analysis between CD209, MRC1, UGT2B4, KRT5, BIRC3, FABP7, and KLRB1 and fatty acid synthesis (FAS) pathway scores. Ferroptosis scores were calculated using ssGSEA based on ferroptosis marker genes from the FerrDb V2 database. FAO and FAS pathway scores were calculated using ssGSEA based on gene sets from the MSigDB database. Analyses were conducted in HR+ breast cancer samples from the TCGA cohort.

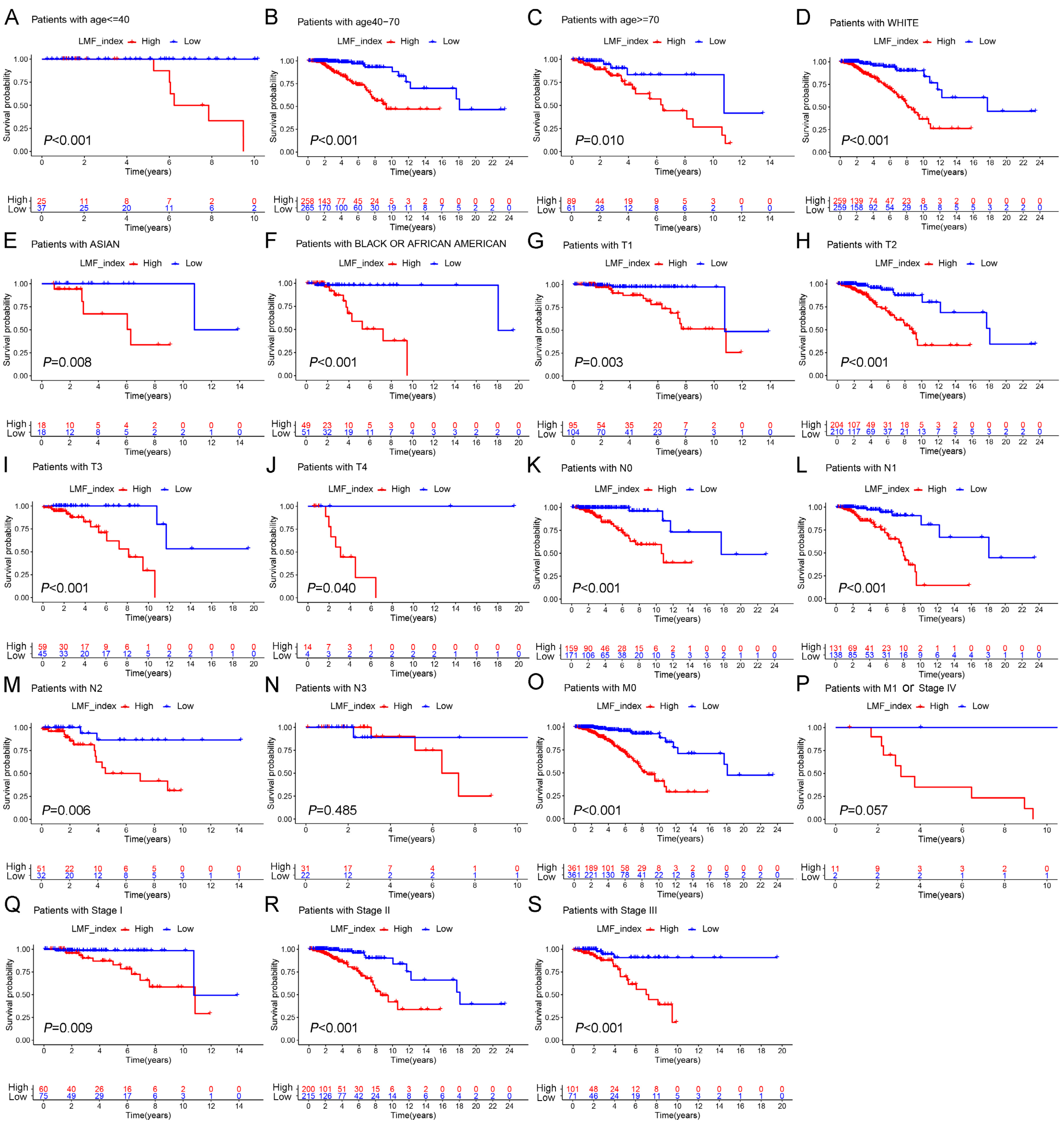

Figure S6. Subgroup Kaplan-Meier survival analysis of clinical pathological characteristics.

(A-S) Kaplan-Meier overall survival (OS) analysis for patients stratified by age (≤40 years, 40－70 years, ≥70 years), race (White, Asian, Black or African American), tumor stage (T1, T2, T3, T4), nodal status (N0, N1, N2, N3), metastatic status (M0, M1), and disease stage (Stage I, Stage II, Stage III, Stage IV).

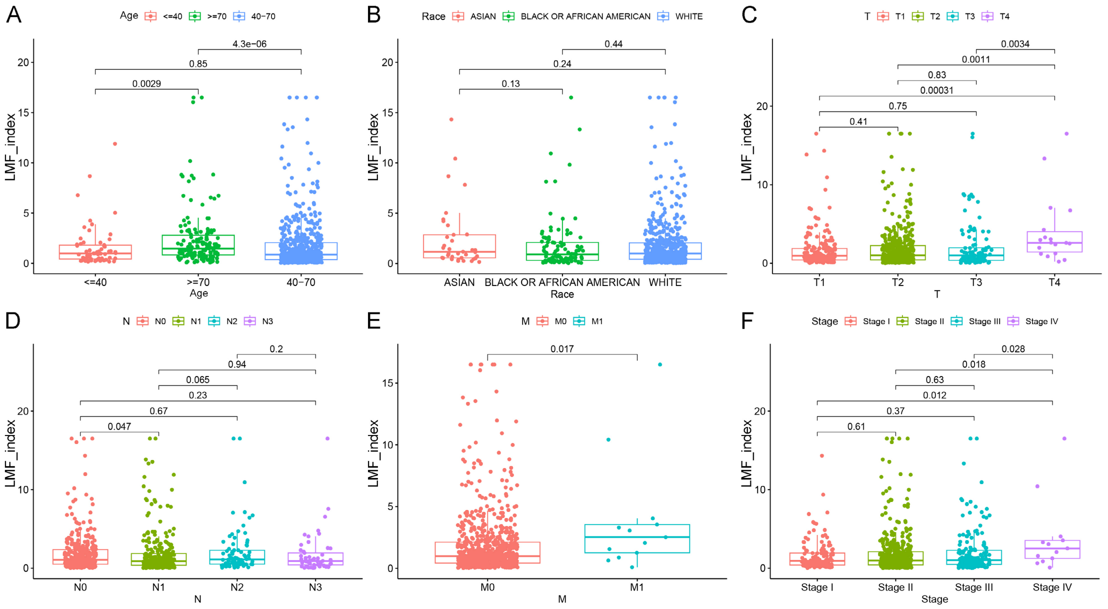

Figure S7. Differential analysis of LMF_index across various clinicopathological features.

(A-F) Comparison of risk scores among different age groups, race, T stages, N stages, M stages, and disease stages.

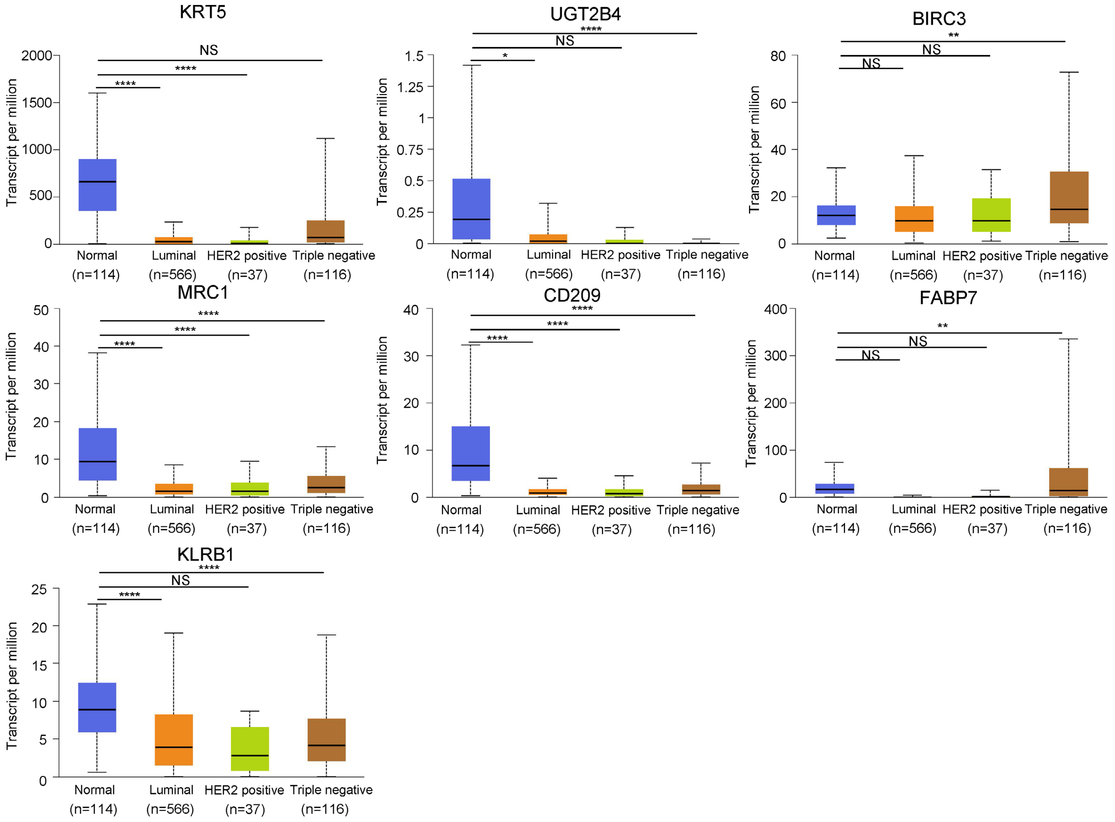

Figure S8. Analysis of expression levels of LMF_index panel genes using the UALCAN database.

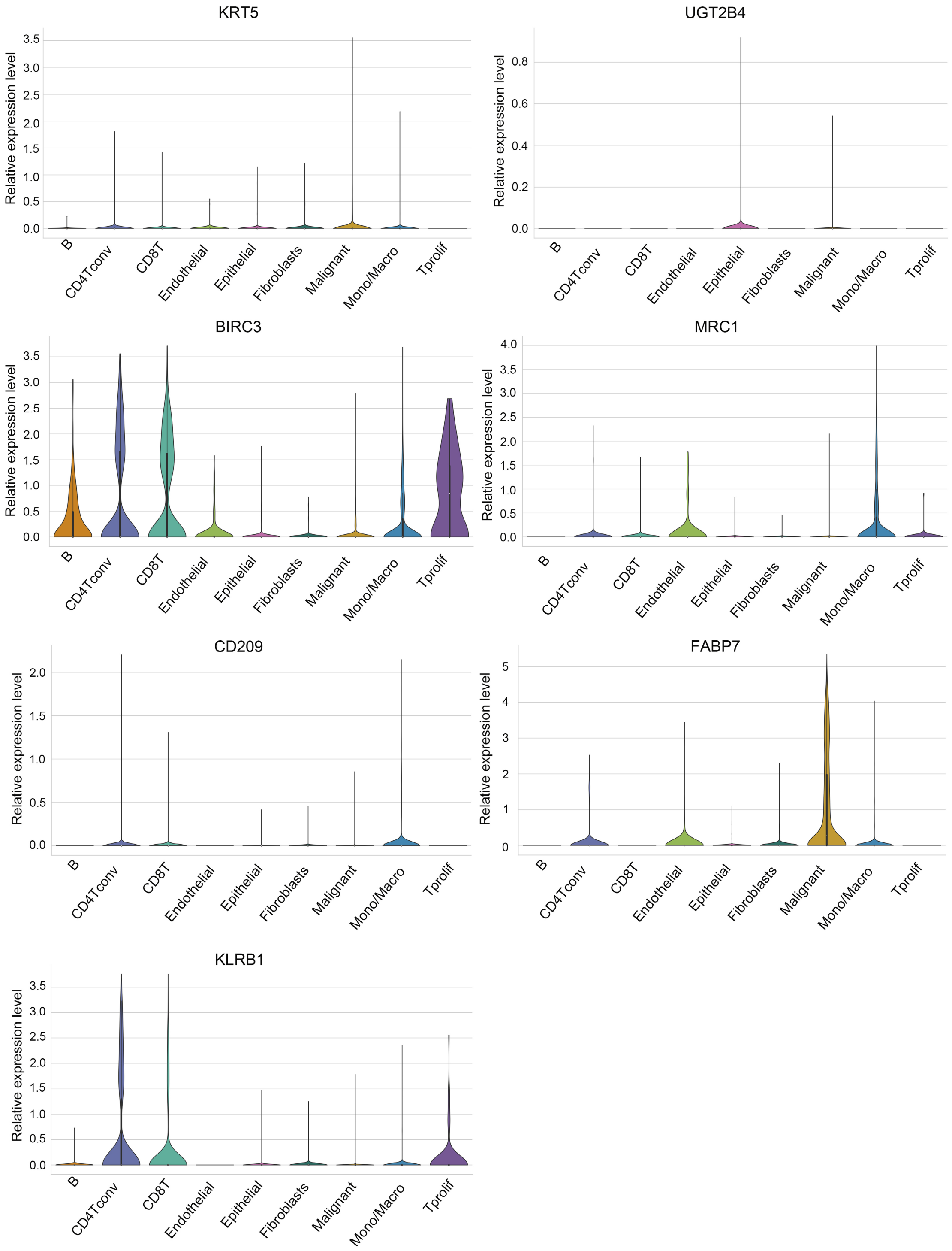

Figure S9. Relative expression levels of LMF_index panel genes in different immune and tumor cell types, related to Figure 8.

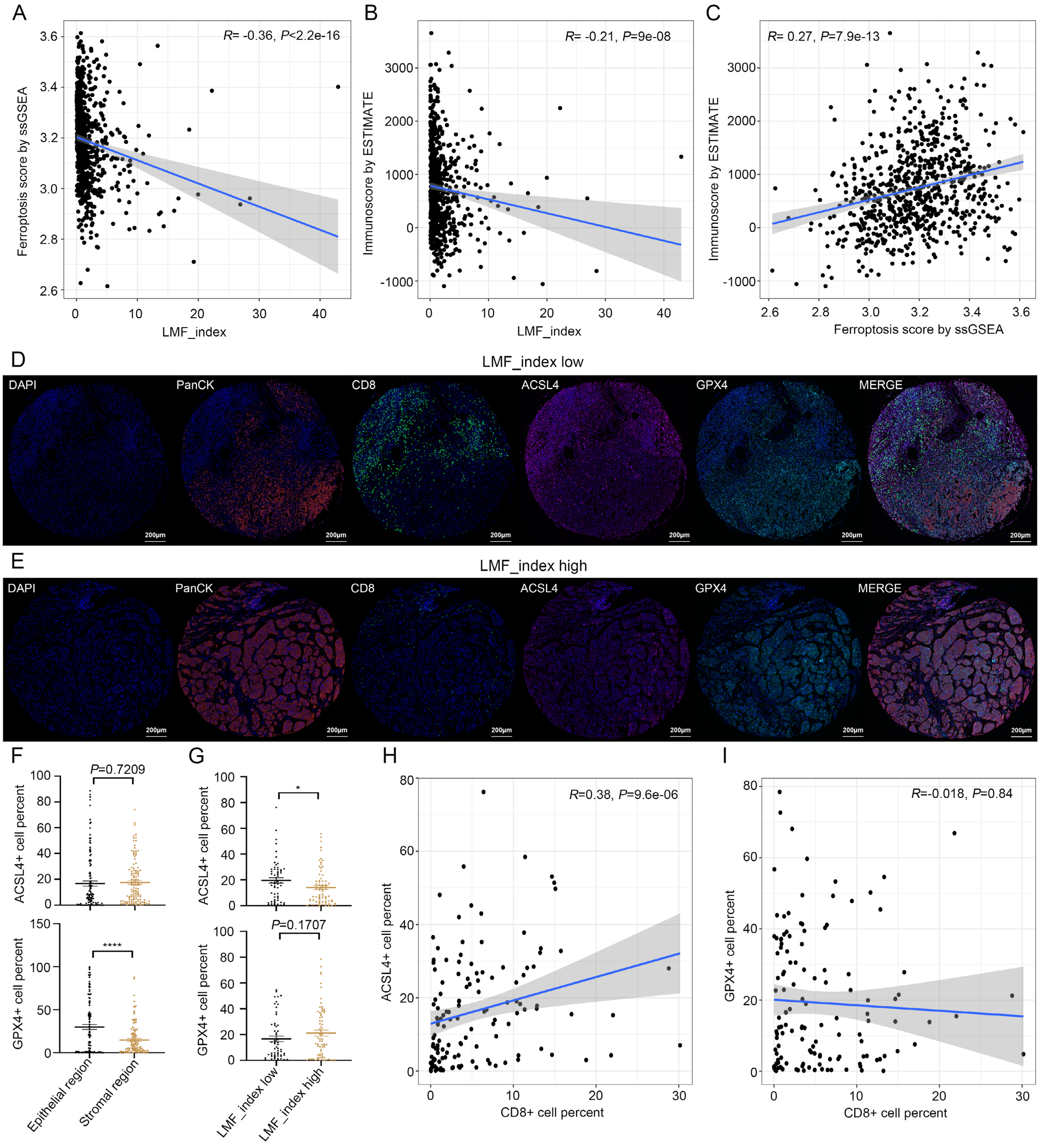

Figure S10. Correlation of LMF_index with ferroptosis and immune infiltration, and validation through multiplex immunohistochemistry, related to Figure 9.

(A) Spearman correlation analysis between the LMF_index and ferroptosis scores calculated by ssGSEA using ferroptosis marker genes from the FerrDb V2 database in hormone receptor-positive (HR+) breast cancer samples from the TCGA cohort. **(B)** Spearman correlation analysis between the LMF_index and immunoscores derived from the ESTIMATE algorithm in HR+ breast cancer samples from the TCGA cohort. **(C)** Correlation analysis between ferroptosis scores and immunoscores in HR+ breast cancer samples. **(D-E)** Representative multiplex immunohistochemistry (mIHC) images showing the spatial expression patterns of PanCK, CD8, ACSL4, and GPX4 in LMF_index low and high groups. **(F)** Quantification of ACSL4+ and GPX4+ cells in epithelial versus stromal regions. **(G)** Quantitative comparison of ACSL4+ and GPX4+ cell percentages between LMF_index low and high groups. **(H-I)**

Spearman correlation between CD8+ T cell infiltration and ACSL4+ or GPX4+ cell percentages in breast cancer tissue microarrays. *$P < 0.05$; ****$P < 0.0001$.

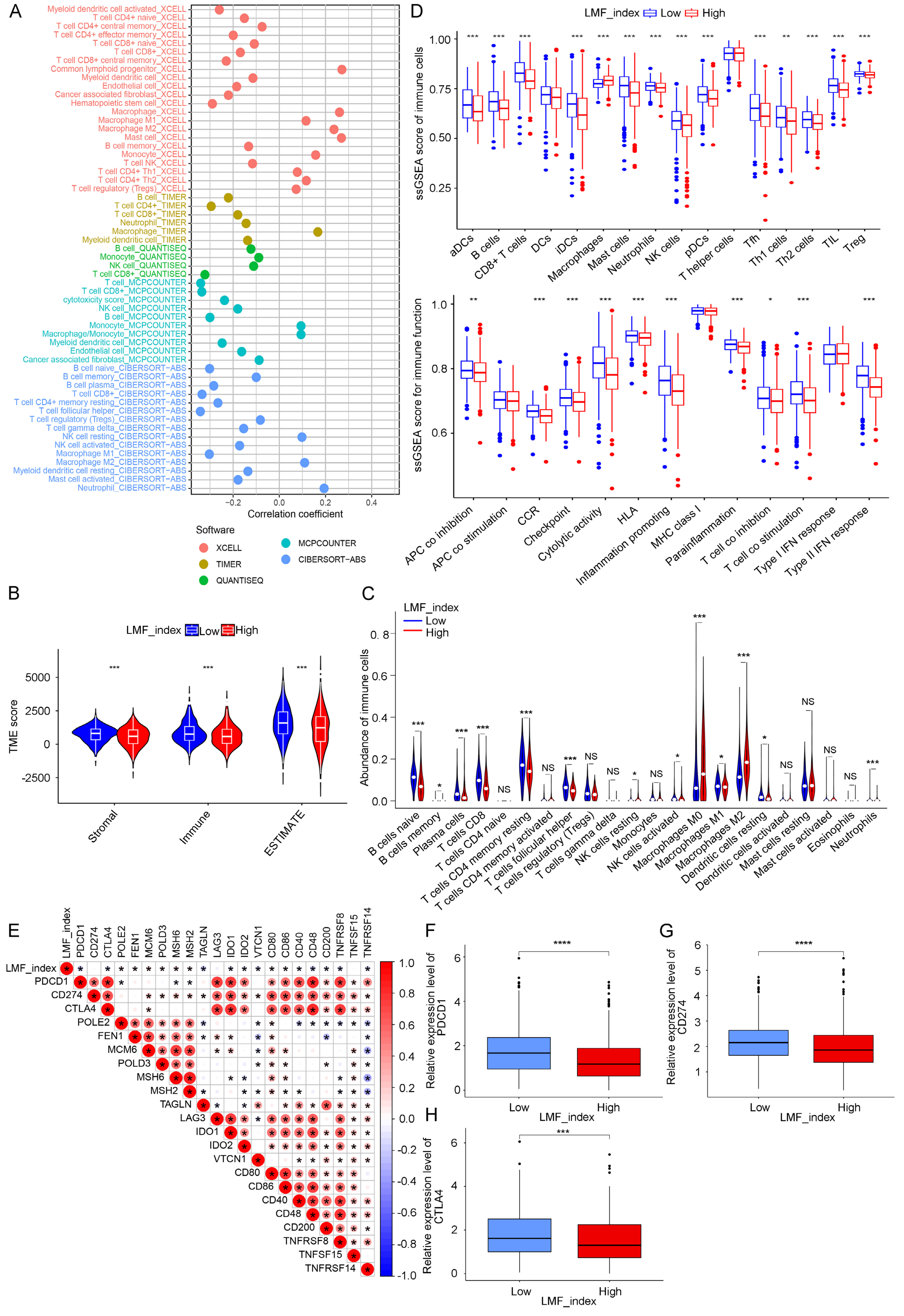

Figure S11. Immune landscape analysis in HR+ breast cancer, related to Figure 9.

(A) Spearman correlation analysis of immune cell infiltration abundance and LMF_index in HR+ breast cancer using multiple algorithms. **(B)** Analysis of stromal scores, immune scores, and ESTIMATE scores across different LMF_index groups. **(C)** Immune cell infiltration abundance across different LMF_index groups using the CIBERSORT algorithm. **(D)** Immune cell infiltration levels and activity of immune-related functions in different LMF_index groups using ssGSEA. **(E)** Heatmap showing the correlation between LMF_index and immune checkpoint-related gene expression. **(F-H)** Differential expression analysis of PD-1, PD-L1, and CTLA4 between LMF_index low and high groups. *$P < 0.05$; **$P < 0.01$; ***$P < 0.001$; ****$P < 0.0001$.

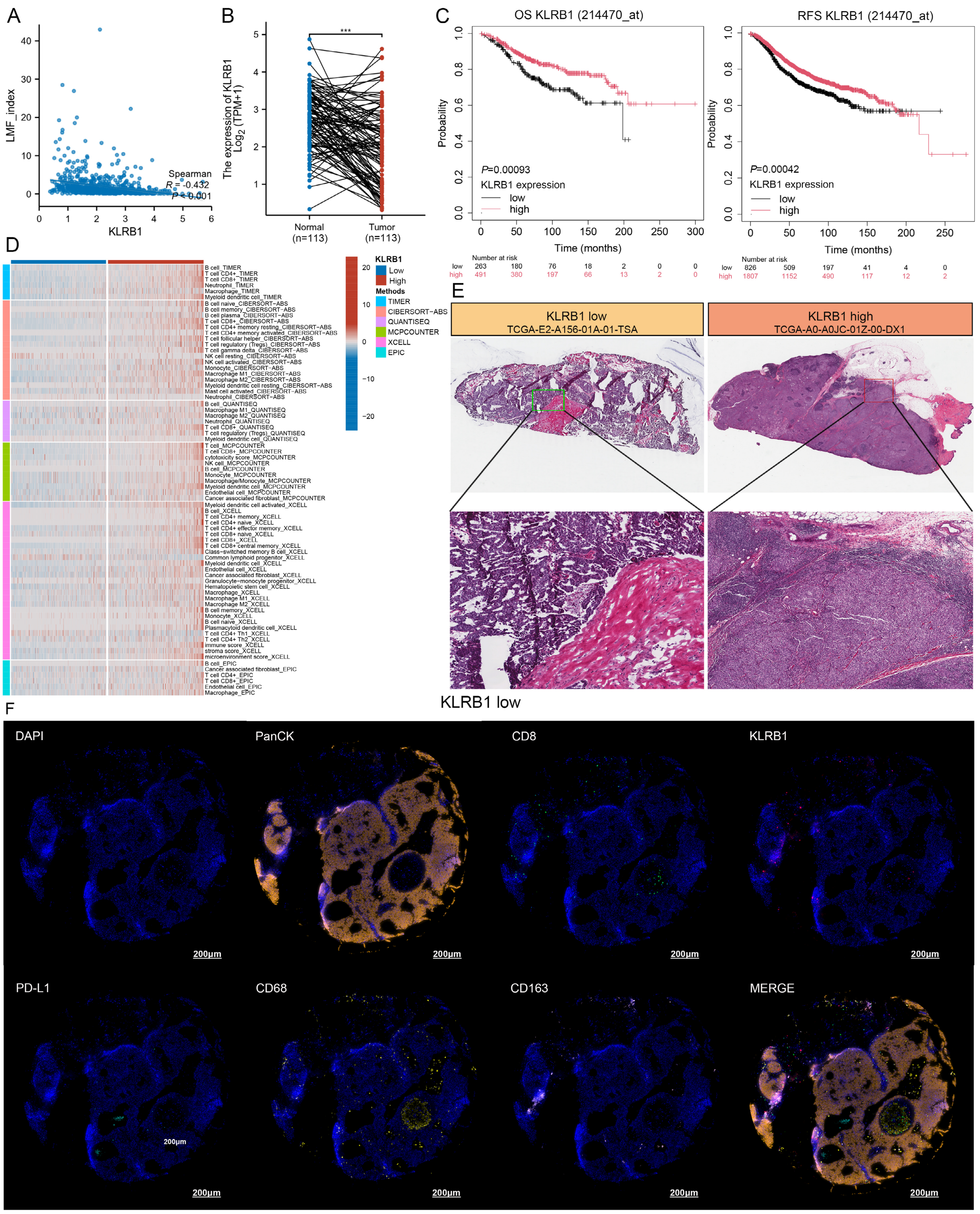

Figure S12. High expression of KLRB1 in HR+ breast cancer is associated with favorable prognosis and immune cell infiltration, related to Figure 10.

(A) Spearman correlation analysis between KLRB1 expression levels and LMF_index. **(B)** Expression analysis of KLRB1 in paired normal breast tissue and breast cancer samples. **(C)** Kaplan-Meier overall survival (OS) and recurrence-free survival (RFS) analyses based on KLRB1 expression levels in HR+ breast cancer patients from the Kaplan-Meier plotter database. **(D)** Analysis of the relationship between KLRB1 expression levels and immune cell infiltration in HR+ breast cancer based on multiple algorithms. **(E)** Comparison of lymphocytic infiltration levels in hematoxylin and eosin (H&E) staining between high and low KLRB1 expression groups. **(F)** Representative images of PanCK, CD8, KLRB1, PD-L1, CD68, and CD163 based on multiplex immunohistochemistry (mIHC) staining in the KLRB1 low group.
